# Following in Emil Fischer’s Footsteps: A Site-Selective Probe of Glucose Acid–Base Chemistry

**DOI:** 10.1021/acs.jpca.1c04695

**Published:** 2021-07-30

**Authors:** Sebastian Malerz, Karen Mudryk, Lukáš Tomaník, Dominik Stemer, Uwe Hergenhahn, Tillmann Buttersack, Florian Trinter, Robert Seidel, Wilson Quevedo, Claudia Goy, Iain Wilkinson, Stephan Thürmer, Petr Slavíček, Bernd Winter

**Affiliations:** † Fritz-Haber-Institut der Max-Planck-Gesellschaft, Faradayweg 4-6, 14195 Berlin, Germany; ‡ Department of Physical Chemistry, University of Chemistry and Technology, Technická 5, Prague 6 16628, Czech Republic; § Institut für Kernphysik, Goethe-Universität, Max-von-Laue-Straße 1, 60438 Frankfurt am Main, Germany; ∥ Operando Interfacial Photochemistry, Helmholtz-Zentrum Berlin für Materialien und Energie, Albert-Einstein-Straße 15, 12489 Berlin, Germany; ⊥ Institut für Chemie, Humboldt-Universität zu Berlin, Brook-Taylor-Str. 2, 12489 Berlin, Germany; # Centre for Molecular Water Science (CMWS), Photon Science, Deutsches Elektronen-Synchrotron (DESY), Notkestraße 85, 22607 Hamburg, Germany; ▽ Department of Locally-Sensitive & Time-Resolved Spectroscopy, Helmholtz-Zentrum Berlin für Materialien und Energie, Hahn-Meitner-Platz 1, 14109 Berlin, Germany; ○ Department of Chemistry, Graduate School of Science, Kyoto University, Kitashirakawa-Oiwakecho, Sakyo-Ku, Kyoto 606-8502, Japan

## Abstract

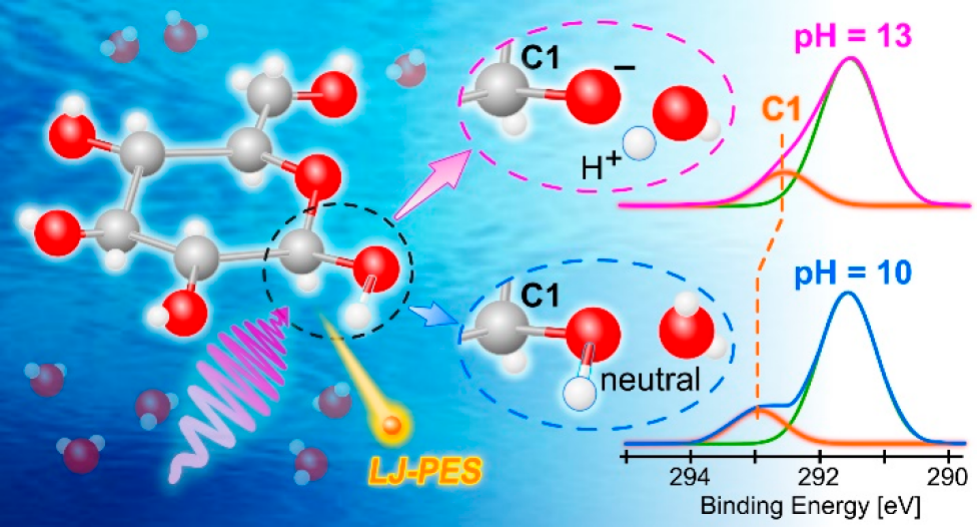

Liquid-jet photoelectron spectroscopy was applied to determine the first acid dissociation constant (p*K*_a_) of aqueous-phase glucose while simultaneously identifying the spectroscopic signature of the respective deprotonation site. Valence spectra from solutions at pH values below and above the first p*K*_a_ reveal a change in glucose’s lowest ionization energy upon the deprotonation of neutral glucose and the subsequent emergence of its anionic counterpart. Site-specific insights into the solution-pH-dependent molecular structure changes are also shown to be accessible via C 1s photoelectron spectroscopy. The spectra reveal a considerably lower C 1s binding energy of the carbon site associated with the deprotonated hydroxyl group. The occurrence of photoelectron spectral fingerprints of cyclic and linear glucose prior to and upon deprotonation are also discussed. The experimental data are interpreted with the aid of electronic structure calculations. Our findings highlight the potential of liquid-jet photoelectron spectroscopy to act as a site-selective probe of the molecular structures that underpin the acid–base chemistry of polyprotic systems with relevance to environmental chemistry and biochemistry.

## Introduction

Glucose is a ubiquitous monosaccharide of major significance in living organisms.^1,2^ It is the precursor of many oligo- and polysaccharides that mediate cell–cell communication,^3^ build up the scaffold of cells,^4−6^ or serve as energy storage units.^7−10^ It is a natural energy source synthesized via the conversion of solar energy into chemical energy by plants during photosynthesis.^11,12^ Consequently, it plays a central role in the metabolic pathways that govern the flow of energy and matter that sustain life.^13^ As a result, it has also become relevant in the investigation of renewable energy technologies that seek to mimic nature, in particular, with the demonstration of alkaline glucose fuel cells.^14,15^

The chemistry associated with the use of glucose as a fuel source, in both living organisms and technological devices, is inherently related to the nature of its structure–function relationship and acid–base chemistry in aqueous solution. Despite this, there remains much to be learned about the acid–base properties of this fundamental molecule. This is perhaps surprising because the structure of glucose has been intensively studied since the turn of the 19th century, when Emil Fischer (Nobel Prize in Chemistry 1902) reported the chemical synthesis of d-(+)-glucose and demonstrated its stereoisomeric forms.^16,17^ But it is only with advancing experimental and theoretical methods,^18−21^ in particular, photoelectron spectroscopy (PES) from an aqueous solution,^22−25^ that previously inaccessible molecular structural details can now be resolved.

With an elemental composition of C_6_H_12_O_6_, glucose is an aldohexose with an aldehyde group at the C1 position in the Fischer projection. In aqueous solution, glucose predominantly adopts the six-membered closed-ring pyranose structure (>99%), in which the C1 atom forms a hemiacetal linkage to the C5 atom. In smaller quantities, the five-membered, closed-ring furanose (<0.5%) and linear (<0.05%) forms are also present.^26,27^ Traditionally, the stereochemistry in glucose molecules is denoted by the relative orientation of the hydroxyl group at the C5 site, which points to the left in the L form and to the right in the D form, as viewed in the Fischer projection. As with many biomolecules, such as amino acids, proteins, and DNA, glucose exhibits a remarkable degree of homochirality. In nature, the right-handed D form dominates.^28,29^ Upon cyclization into the pyranose form, a new stereocenter emerges at the C1 site. At this anomeric center, the stereochemistry is denoted depending on the relative orientation of the hemiacetal hydroxyl group as α or β. Because there are multiple stereocenters within the molecule, α- and β-glucose are diastereomers, which exhibit different physicochemical properties. In solution, the α and β forms of glucose are in equilibrium with the open-ring form and therefore constantly interconvert through a process known as mutarotation,^30^ with β-glucose being favored (∼38% α-glucose vs ∼62% β-glucose) due to the anomeric effect;^27,31^ however, this equilibrium can be shifted by tuning the solution pH^32^ or through the addition of inorganic salts.^33^ Glucose is also known to readily isomerize under alkaline conditions to fructose and mannose via Lobry de Bruyn–Alberda van Ekenstein transformations, a process of significance in the preparation of liquid hydrocarbon fuels.^34,35^

**Figure 1:**
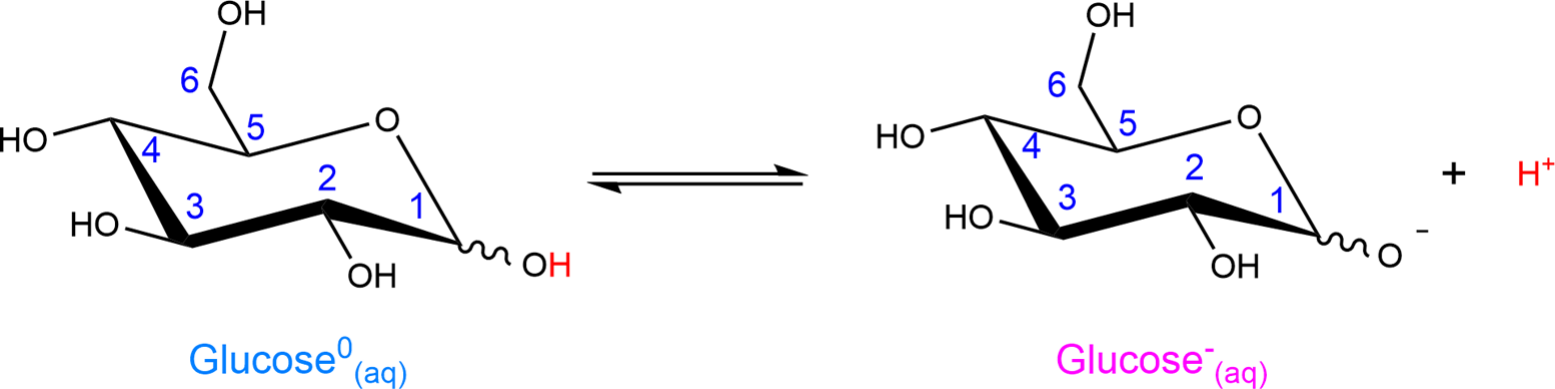
Schematics of the predominantly adopted six-membered closed-ring protonated (glucose^0^_(aq)_) and deprotonated (glucose^–^_(aq)_) structures of glucose in aqueous solution.^40,42^ C–OH sites are labeled 1–6, and the nomenclature is used throughout the text. The zigzag line is used to indicate the two possible orientations of the hydroxyl group at the C1 site, downward or upward, in the α and β anomeric forms, respectively.

Glucose is a weak acid with at least two reported acid-ionization (*i.e*., deprotonation) equilibria and acidity constants (p*K*_a_) of 12.1 (p*K*_a1_) and 13.9 (p*K*_a2_),^36,37^ which highlight its enhanced reactivity in alkaline media.^38,39^ Previous determinations of glucose p*K*_a_ values involved the use of site-insensitive high-performance liquid chromatography^39^ and titration-based methods.^36,40,41^ Consequently, ambiguity remains regarding the extent to which the transition defined at p*K*_a1_ involves charge sharing with C–OH groups beyond the C1 site.^42^ Schematic representations of both the neutral (glucose^0^_(aq)_) and deprotonated (glucose^–^_(aq)_) forms^40,42^ of aqueous glucose are shown in Figure 1.

Whereas site-sensitive methods such as X-ray PES have been applied to study glucose in the solid phase,^43−45^ this approach is unable to access the behavior of aqueous-phase glucose, which is expected to be significantly affected by intra- and intermolecular hydrogen bonding.^32,40,46^ In this context, a local, site-selective, solution-phase characterization of the molecular structure of glucose is needed to better understand its acid–base behavior. To meet this challenge, we leverage the unique experimental capabilities of liquid-jet PES (LJ-PES),^22,47^ which enable us to probe the overall and local electronic structure of protonated and deprotonated solvated glucose via valence and site-specific measurements, respectively. This approach has been previously applied to investigate pH-dependent electronic-structure changes and protonation sites in amino acids.^48,49^

Herein, we present a combined experimental and theoretical study of the molecular structure of glucose in the pH range from 2 to 13. Utilizing soft X-ray LJ-PES, we monitor (mostly alkaline) pH-dependent changes in the solution-phase valence and C 1s PES spectra of glucose. Specifically, we probe aqueous-phase binding energy (BE) shifts associated with the most acidic C–OH group(s) and determine the p*K*_a1_ value. To gain insight into the existence of single or multiple deprotonation equilibria related to p*K*_a1_, we investigate the associated chemical structures and confirm principal spectral assignments with the aid of electronic-structure calculations. Our work highlights LJ-PES as a methodology to site-selectively probe ionization equilibria in solvated species, in contrast with previous site-unspecific measurements.

## Methods

### Experiments

Glucose aqueous solutions of 1 M concentration were prepared at room temperature by dissolving α-d-(+)-glucose crystals (Acros Organics, >99% purity, anhydrous) in Millipore water (55 nS/cm). Measurements were performed from both freshly prepared and several-hour-old solutions, yielding identical spectra and implying that α and β anomers cannot be distinguished in the present experiments. The pH was varied by the dropwise addition of either HCl (10% and 37% w/w) or NaOH (1 M and 10 M) aqueous solutions under constant magnetic stirring. The solution pH was monitored with a pH meter (VWR, pHenomenal 1100L) to produce 1 M samples at pH values of 2.0, 7.4, 10.0, 10.5, 11.0, 11.5, 12.0, 12.5, and 13.0. Glucose aqueous solutions of 1 M concentration without prior pH adjustment were found to exhibit a pH of 4.8 ± 0.2. Because of the protonated, nonionic nature of pure glucose, NaCl (Aldrich, ≥99%) in 25–50 mM concentration was added to those samples to ensure sufficient solution conductivity and minimize sample charging due to streaming potentials.^50^ LJ-PES experiments were performed at the U49/2 PGM-1 beamline^51^ at BESSY II using the SOL^3^PES setup^52^ and the P04 beamline^53^ at PETRA III using the EASI setup.^54^ Although the majority of the measurements were conducted at the P04 beamline, spectra from glucose solutions at two complementary pH values (11 and 12) were subsequently recorded at BESSY II. The latter yielded somewhat lower electron count rates due to the lower photon flux. At BESSY II, experiments were performed with horizontally polarized light, whereas P04 provided circularly polarized light. Both setups were equipped with differentially pumped hemispherical electron analyzers that detected photoelectrons emitted from the sample either at 0° with respect to the light polarization (SOL^3^PES) or at 50° with respect to the light propagation (EASI). The samples were introduced into the experimental chamber in the form of liquid microjets^55^ using glass capillary nozzles of 25–35 μm inner diameter and 0.6–0.8 mL/min sample flow rates. In the SOL^3^PES setup, the (vertical) liquid-jet flow direction was orthogonal with respect to the light polarization and the electron analyzer detection axes. In the EASI setup, the (horizontal) liquid-jet flow was at 90° with respect to both the light propagation (floor plane) and the electron detection (at an angle of 50° to the floor plane) directions. The liquid jet was electrically connected and grounded to the experimental setup by means of a gold wire immersed in the electrically conductive solution (in the case of SOL^3^PES) or by a small metallic tube inserted into the main polyether ether ketone (PEEK) liquid delivery line (in the case of EASI). Valence and C 1s spectra were recorded as a function of pH using photon energies of 600 eV and 850 eV, respectively. Such photon energies enabled us to produce photoelectrons with approximately 590 eV (valence) and 560 eV (C 1s) kinetic energies, thus ensuring sufficiently large probing depths into the liquid jet^56^ (*i.e*., probing of fully hydrated glucose molecules). At PETRA III, the overall experimental energy resolution was 230 meV (for 600 eV photon energy) and 280 meV (for 850 eV photon energy), respectively. The complementary measurements at BESSY II had a somewhat lower overall resolution of 380 meV (850 eV only).

### Computations

Valence vertical ionization energies (VIEs), that is, BEs, were calculated for glucose structures optimized at the density functional theory (DFT) level using the CAM-B3LYP^57^ functional and the 6-31+G* basis set. All of the optimized structures were confirmed as energy minima via frequency analysis. The polarizable continuum model (PCM)^58,59^ was used to mimic the presence of the water solvent. In particular, the nonequilibrium PCM^60^ was used to describe the fast photoelectron ionization process. To account for specific intermolecular interactions, several nearest water molecules were considered explicitly. Calculations were performed in Gaussian 09 (revision D.01)^61^ using the default parameters for the PCM. VIEs were calculated with the DFT-based delta self-consistent field (ΔSCF) approach, followed by a time-dependent DFT (TDDFT) evaluation of the BEs for deeper-lying electrons. The VIEs were then calculated as^62^
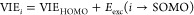
where VIE_*i*_ is the VIE of the *i*th electron and *E*_exc_ is the excitation energy, restricted to excitations into the singly occupied molecular orbital (SOMO).

Core-level VIEs were calculated using the maximum overlap method (MOM)^63^ in the Q-Chem 4.3 software.^64^ This approach is centered on ground-state electronic structure techniques while avoiding the variational collapse of the wave function and providing reliable core-electron BEs for solvated systems.^65^ Calculations were performed using the CAM-B3LYP functional and the cc-pVTZ split basis set for all of the H atoms in glucose. For the C and O atoms, the aug-cc-pCVTZ basis set was used. The parameters in the nonequilibrium PCM were set to match those in Gaussian 09, revision D.01^61^ (atomic radii from Universal Force Field^66^ and scaling factor α = 1.1). C 1s PES spectra were modeled from the calculated VIEs at the optimized geometries via the empirical broadening scheme. In that way, each modeled spectrum was calculated considering six energies originating from six C atoms in the molecule, and the corresponding sum of six Gaussian components was centered at the respective VIEs. The width of each Gaussian is characterized by a standard deviation (σ) of 0.45 eV, which is reasonable for PES spectra of solvated systems.^67^ This value was found to fit the experimental PES data of fully protonated glucose (*i.e*., data recorded at pH 10; see Figure 3) and was thus subsequently used as a constant to simulate similar PES spectra for the different deprotonated forms (*i.e*., considering the deprotonation of different hydroxyl groups).

Calculations of p*K*_a_ values were performed using Gaussian 09 (revision D.01)^61^ and implementing two different approaches. The first approach was based on a thermodynamic cycle including the deprotonation of glucose in the gas phase. Within that methodology, aqueous-phase deprotonation energetics (*i.e*., changes in Gibbs free energies, Δ*G*_(aq)_) were calculated by evaluating their gas-phase counterpart (Δ*G*_(g)_) and adding solvation energies (Δ*G*_(solv)_). Thus the energies associated with the solvation of gas-phase reactants (Δ*G*_(solv.,react.)_) and products (Δ*G*_(solv,prod)_) were used to calculate Δ*G*_(aq)_ as
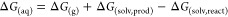
The gas-phase energetics were evaluated using CAM-B3LYP/aug-cc-pVTZ at a temperature of 298.15 K using frequency calculations to obtain the gas-phase free energies. Solvation energies of protonated and deprotonated glucose were calculated using the PCM. The solvation energy of the proton was taken from the literature to be −265.9 kcal/mol.^68^ The second, more straightforward approach consisted of the implementation of the methodology proposed by Thapa and Schlegel.^69^ This method uses a polarizable solvation model to directly evaluate aqueous-phase free-energy changes. According to the authors, reasonable results can be obtained when using a combination of the ωB97XD^70^ method with a 6-31+G* basis set and the solvation model based on solute electron density (SMD).^71^

## Results and Discussion

### Valence PES Spectra from Protonated and Deprotonated Aqueous-Phase Glucose

**Figure 2:**
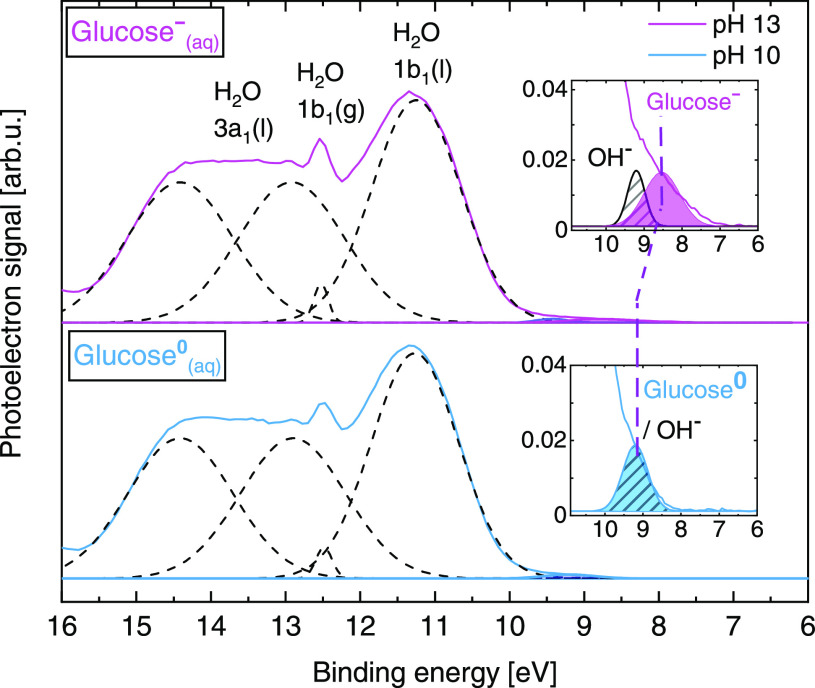
Valence PES spectra of 1 M glucose aqueous solutions at pH 10 (bottom, light blue curve) and 13 (top, magenta curve) measured at a 600 eV photon energy. The dashed lines are Gaussian curves representing signal contributions from water’s leading orbitals, 1b_1_ and (split) 3a_1_; liquid (l) and gas-phase (g) signals are assigned.^72^ Spectra intensities are displayed to yield the same height as the 1b_1(l)_ peak. The figure insets show enlarged views of the photoelectron features associated with the lowest vertical ionizing transitions in glucose^0^_(aq)_ (light blue peak) and glucose^–^_(aq)_ (magenta peak) as well as from OH^–^_(aq)_ (peak with striped fill). The BE of the latter was fixed during the fitting procedure according to the value reported in ref (77).

Valence PES spectra from 1 M glucose aqueous solutions at pH 10 (below p*K*_a1_, bottom) and 13 (above *pK*_a1_, top) are shown in Figure 2. The spectra are representative of protonated (glucose^0^_(aq)_) and deprotonated (glucose^–^_(aq)_) molecules. The data are presented on a BE scale calibrated according to the 1b_1_ BE of neat liquid water.^a^ The spectra are almost identical, dominated by the contributions from liquid water corresponding to the ionization of water’s 1b_1_, (split) 3a_1_, and 1b_2_ leading orbitals (as labeled in Figure 2; see ref (72) for details). Small contributions from gaseous water from the vapor layer surrounding the liquid jet can also be observed (mainly from 1b_1(g)_ photoelectrons; see a somewhat sharper peak at ∼12.5 eV).

We expected that a change in the charge state of glucose upon deprotonation, from glucose^0^_(aq)_ at pH 10 to glucose^–^_(aq)_ at pH 13, would lead to a change in the lowest VIE of the molecule (as demonstrated in previous experiments on the pH-dependent changes in the lowest VIEs of aqueous imidazole,^73,74^ phosphate,^75^ and phenol/phenolate^76^). A small but decisive difference between the pH 10 and pH 13 data is the occurrence of a photoelectron signal near 8.5 eV BE in the pH 13 spectrum. Enlarged views of this spectral region are presented in the figure inset. Gaussian peaks from a cumulative fit analysis highlight the spectral contributions from glucose^–^_(aq)_ (at 8.5 ± 0.3 eV, magenta fill), added hydroxide (OH^–^_(aq)_, striped fill, fixed at 9.2 eV BE, as reported in ref (77)), and glucose^0^_(aq)_ (light blue fill, overlapping with the OH^–^_(aq)_ feature). Note that lifetime broadening effects are negligible in the case of valence ionization, and a cumulative Gaussian fit approach is thus appropriate. The overall error is a combination of the fitting error reported from the least-squares fitting procedure, the experimental resolution (from both the light source and the electron analyzer) of ∼0.23 eV, and the error associated with the calibration of the BE scale. As previously stated, the BE scale is referenced to the 1b_1_ BE of neat liquid water, which, however, may deviate slightly from the 1b_1_ BE value of the solution. Solute-induced changes in the electronic structure of water and the work function of the solution may occur, which shift the BE of the photoelectron features. A slight shift of the liquid 1b_1_ peak for very high (up to 8 M) concentrations of NaI has been discussed lately by some of the coauthors^78^ and is being further investigated by our group. Notably, we do not expect any detectable reference-level changes from the 1 M glucose solute, similarly from the small amounts of HCl_(aq)_ and NaOH_(aq)_ added to the solutions. On this basis, we estimate cumulative valence peak BE errors of 0.3 eV. Our interpretation of the spectral changes observed upon changing the solution pH from 10 to 13 was corroborated by our calculations, which revealed glucose^–^_(aq)_ to be the prevalent species at pH 13, as opposed to glucose^0^_(aq)_ at pH 10, as shown in the following.

We have evaluated the respective valence VIEs for the minimum-energy structures of protonated and deprotonated glucose in both the α and β anomeric forms. The calculated structures are consistent with the energetic minima previously published,^79,80^ showing only minor energy differences between the α and β forms for both gas- and aqueous-phase glucose. (See Table S1 in the Supporting Information (SI) for details.) Because of the very high charge density at the O atom, the addition of explicit solvent molecules is required to reach quantitatively correct values in calculations of the electron BEs for glucose^–^_(aq)_. (The calculated results are not sensitive to the explicit solvent addition for glucose^0^_(aq)_.)^81^ The highest occupied molecular orbital (HOMO), associated with the lowest VIE, is of π character and is delocalized over the whole molecule for neutral glucose^0^_(aq)_. However, the HOMO is more localized on the single C and single O of the deprotonated hydroxyl group in glucose^–^_(aq)_.

The first VIE of fully protonated glucose (glucose^0^_(aq)_), with the explicit inclusion of close-range water interactions represented by two water molecules, was calculated to be 9.09 eV (Table 1), in good agreement with the onset of the spectrum at pH 10. The solvent shift (*i.e*., the difference between the gas- and solution-phase VIEs) is ∼1 eV. For glucose^–^_(aq)_, the calculated first VIE is at 7.95 eV (using six explicit water molecules; the larger number of explicit solvating molecules is needed due to a slower BE convergence compared with the neutral species; see Table S1 in the SI), >1 eV lower than that of glucose^0^_(aq)_, in reasonable agreement with the 8.5 eV feature observed from the experimental data recorded at pH 13. (A solvent shift of almost 2 eV is observed in this case.) Note that the calculated VIEs without the inclusion of specific water interaction are much lower (6.75 eV). The VIEs for the various deprotonation sites and for the different anomers are rather close in energy, as presented in Table S1 in the SI for both α and β anomers.

**Table 1:** Calculated First Three Vertical Ionization Energies (VIEs) of Fully Protonated (glucose^0^_(aq)_) and Singly Deprotonated (glucose^–^_(aq)_) Aqueous-Phase Glucose in Electronvolts^a^

	glucose^0^_(aq)_	glucose^–^_(aq)_
1. VIE (HOMO)	9.09	7.95
2. VIE (HOMO–1)	9.40	8.87
3. VIE (HOMO–2)	9.66	9.44

a Calculations were performed by applying the hybrid model with two or six explicitly solvating water molecules for glucose^0^_(aq)_ and glucose^–^_(aq)_, respectively.

Overall, we conclude that the valence PES spectrum recorded at pH 13 (*i.e*., above p*K*_a1_) reflects the acid ionization of glucose’s hydroxyl groups. However, site-specific insights into the molecular structural changes that take place upon deprotonation, that is, which C–OH groups are involved in the deprotonation equilibria, cannot be inferred from the valence spectra. The reason is that the valence energies of the different hydroxyl groups are too similar to be resolved, and the signal contributions from the solvent as well as the OH^–^_(aq)_ solution component overlap with the primary feature of interest. Site-specific and more differential information is revealed in the C 1s core-level PES spectra, which thus serve as a probe of more specific acid–base properties of glucose, as will be presented in the following.

### C 1s Core-Level PES Spectra from Aqueous-Phase Glucose: pH-Dependent Changes

**Figure 3:**
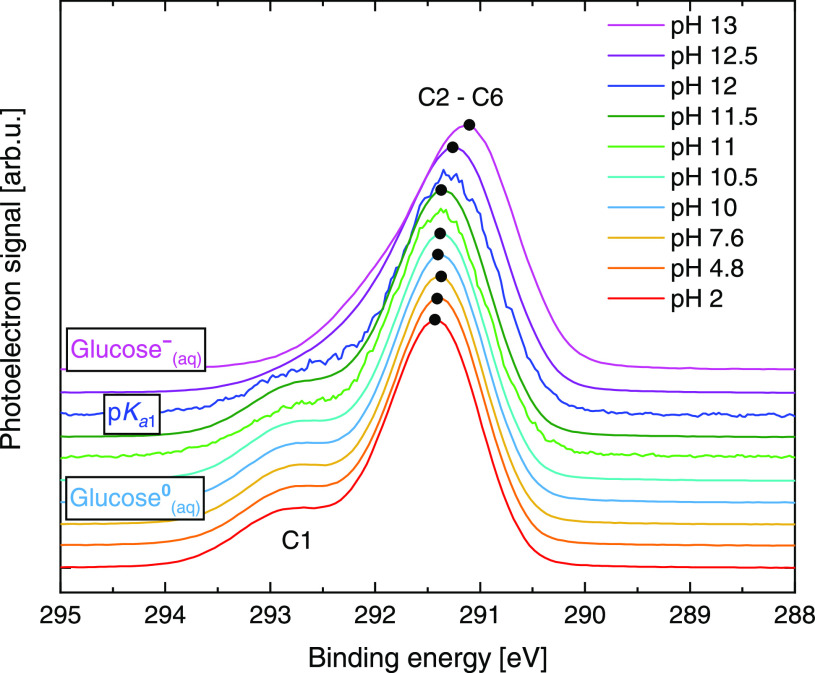
C 1s PES spectra from 1 M glucose aqueous solutions, with pH ranging from 2 to 13, measured at an 850 eV photon energy. The spectra are scaled to yield the same height of the 291.5 eV BE peak and are additionally presented with a vertical offset to highlight the pH-dependent spectral changes. The black dots at the peak of each spectrum serve as a guide for the energy change of the main (C2–C6) peak as a function of pH. Spectra at pH 11 and 12 were measured at the lower-photon-flux beamline and correspondingly have lower signal-to-noise levels. (See the Methods section.) These spectra are manually shifted such that the respective C2–C6 peak centers match the trend of peak maxima (highlighted using black dots).

Figure 3 shows C 1s PES spectra from 1 M glucose aqueous solutions in the 2–13 pH range. As in Figure 2, the data are presented on a BE scale, but this time they are calibrated based on the liquid water O 1s BE^82^ measured from each solution.^a^ In this way, (pH-dependent) sample surface-charging effects are canceled out, and the observed small energy shifts (up to ∼300 meV; see the black dots at the peak of each spectrum to guide the eye) of the overall C 1s spectra are argued to reflect true pH-dependent BE changes, as similarly observed and confirmed by our calculations, see the next section. We further stress that the amounts of HCl_(aq)_ and NaOH_(aq)_ solutes added for pH adjustment are too small to lead to a detectable change in the electronic structure of water.^78^

With reference to available PES studies from solid-phase (crystalline) glucose^43^ and carbon spheres grown from glucose solutions,^44^ the glucose C 1s aqueous-phase spectral features can be crudely assigned to ionization from the C1 and C2–C6 atoms, as labeled in Figure 3. In the 2–10 pH range, we observe a main C 1s peak near 291.5 eV BE accompanied by a higher energy shoulder near 293.0 eV, with no noticeable pH-dependent spectral changes. The latter implies the (near-sole) existence of a single-charge-state species, that is, neutral glucose (glucose^0^_(aq)_) up to pH 10. At pH values higher than 10, the higher energy shoulder becomes less clear, as it appears to move toward lower BEs, consistent with observations from the valence data presented in the previous section. This is indicative of an increase in the fraction of deprotonated glucose molecules up to pH 13 (above p*K*_a1_), when glucose^–^_(aq)_ becomes the prevalent species. (Considering a p*K*_a2_ of 13.9,^36,37^ a fraction of doubly ionized molecules should also be present.)

Qualitatively, the larger C 1s BE of the higher-energy shoulder in glucose^0^_(aq)_ with respect to glucose^–^_(aq)_ reflects the additional positive charge at the specific C site in the former. The observations described here highlight pH-dependent, C-site-specific spectral changes in the C 1s PES data, that is, a site-selective probe of molecular structure changes in glucose upon deprotonation. Details regarding the identity of the C–OH groups involved in the acid dissociation process highlighted in the data are provided by our calculations, as presented in the following section.

### Assignment of the C 1s PES Spectrum of Glucose: Electronic Structure Theory Calculations

The C 1s BEs of each individual C atom (C1–C6) were evaluated using the MOM method combined with the CAM-B3LYP/aug-cc-pCVTZ approach. The BEs were calculated in the gas and aqueous phases for both the α- and β-glucose forms, focusing on the C 1s BE changes taking place upon deprotonation. The calculations considered microsolvation of glucose by the addition of a single explicit water molecule; the addition of further water molecules did not lead to any significant change in the BE. The calculated BEs are summarized in Table S2 in the SI. As follows, we will exclusively discuss the results for the β anomer; the results for the α anomer are very similar.

**Figure 4:**
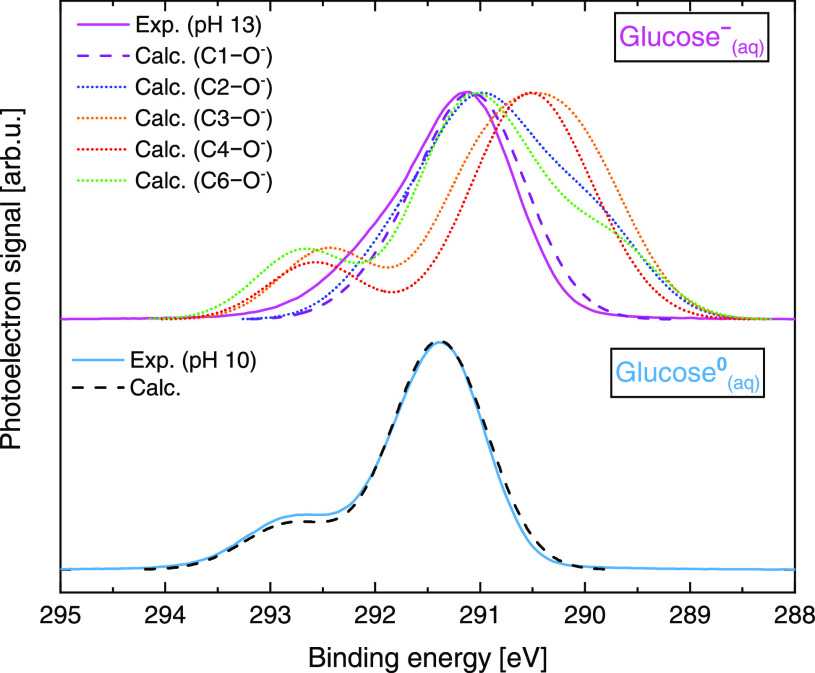
Comparison of experimental (full line) and calculated (dashed and dotted lines) C 1s PES data for glucose^0^_(aq)_ (pH 10, bottom) and glucose^–^_(aq)_ (pH 13, top). All calculated curves were shifted in binding energy by an offset value determined such that the main glucose^0^_(aq)_ peak overlaps with the experimental data at pH 10, as explained in the text. Calculated spectra considering deprotonation at different C sites are shown in the top panel. Note that there is no C5–OH group to deprotonate in the cyclic form.

Simulated C 1s PES spectra for glucose^0^_(aq)_ and glucose^–^_(aq)_ are presented in Figure 4. The corresponding experimental data (C 1s PES spectra recorded at pH 10 and 13, respectively, as shown in Figure 3) are plotted for comparison. For glucose^0^_(aq)_ (bottom panel), the main C 1s feature in the calculated spectra is overlapped with the experimental data. The same spectral shift was applied to the calculated glucose^–^_(aq)_ curves (top panel), which were produced considering deprotonation at different C–OH sites. The BE shift of the C 1s main feature between the experimental and calculated spectra amounted to 240 meV. Such a magnitude accounts for the (expected) few hundred millielectronvolts shift between theory and experiment due to the known shortcomings of DFT calculations in providing core-level BEs on an absolute scale. (We assume that the absolute value is shifted due to the localized nature of the C 1s electron, yet the differences between various forms are faithfully described.) Nonetheless, consistent with the experimental data, our calculations show that all BEs are shifted toward lower values when going from glucose^0^_(aq)_ to glucose^–^_(aq)_. (See Figure 3.)

For glucose^0^_(aq)_, the shape of the calculated, fully protonated (cyclic glucose, pyranose form) spectrum (black dashed line) matches that of the experimental data (light-blue full line). For glucose^–^_(aq)_, a match in spectral shape is observed only with the spectrum calculated considering deprotonation at C1 (purple dashed line). Our calculations show that if we deprotonate any of the other C sites (blue, orange, red, and green dotted lines), then we are left with a separated minor peak at higher energies or a broad shoulder at lower energies. These features are, however, not observed experimentally. Consequently, the experimentally observed higher energy shoulder discussed in the previous section can be safely assigned to the C1 group, whereas the main C 1s feature stems from five close-lying (protonated) components. A more detailed spectral decomposition analysis will be discussed in the next section, with contributions from both theory and experiment.

**Figure 5:**
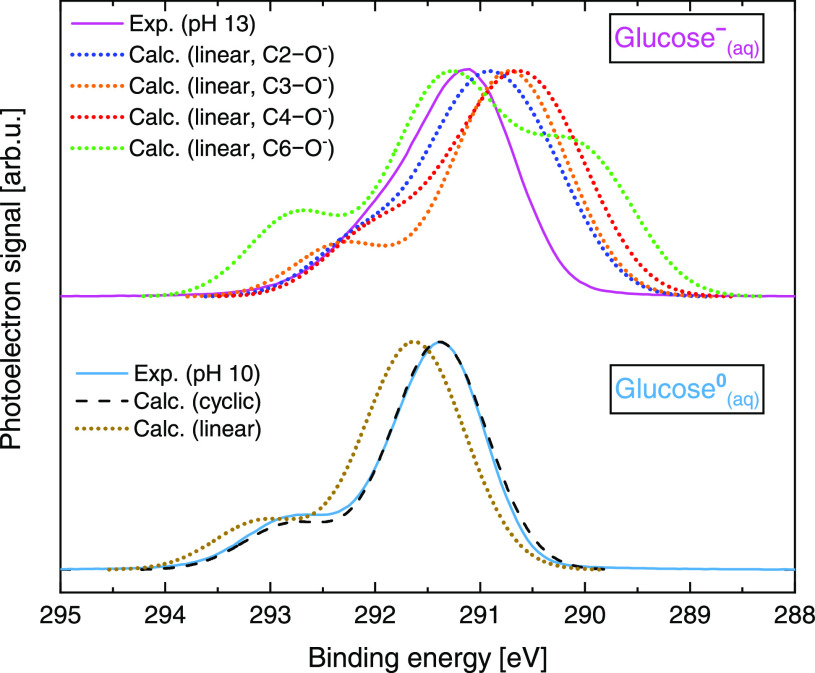
Comparison of experimental (full line) and calculated cyclic and linear (dashed and dotted lines, respectively) C 1s PES spectra of glucose^0^_(aq)_ (pH 10, bottom) and glucose^–^_(aq)_ (pH 13, top). The simulated spectral curves were all shifted by the same value, chosen to overlap the pH 10 data, as explained in the text. Calculated spectra of the linear forms of glucose considering deprotonation at different C sites are shown in the top panel.

Our computations were complemented by quantifying the BEs of the energetically unfavorable noncyclic forms of glucose.^42^ The results are presented in Figure 5, with the computed spectra aligned on the BE scale, as discussed for Figure 4. The absolute calculated BE values are shown in Table S2 in the SI but are not considered in the interpretation of the experimental data because an error on the order of few hundreds of millielectronvolts is expected from DFT-based calculations, as performed here. However, more information can be inferred from the structure of the peak. On the basis of a comparison of experimental and calculated spectral shapes for glucose^0^_(aq)_ (bottom panel), the difference between the cyclic and linear forms is minor. (The linear form has a somewhat larger splitting between the two peaks.) For glucose^–^_(aq)_ (top panel), spectra from linear structures with a deprotonated hydroxyl group at the C3 and C6 sites exhibit rather pronounced shoulders that are not observed experimentally. Note also that spectra corresponding to acid ionization at C2 and C4 are too wide, and the linear structure deprotonated at the C5 position does not represent a stable energy minimum. (Calculations for C1 are not shown because there is no C1–OH group in the noncyclic form.) Furthermore, according to our calculations, noncyclic forms are at least 26 kJ/mol (0.27 eV) higher in energy than the cyclic form, so the relative fraction of noncyclic forms should be <0.003%.

### Spectral Decomposition of the C 1s PES Spectrum of Glucose

**Figure 6:**
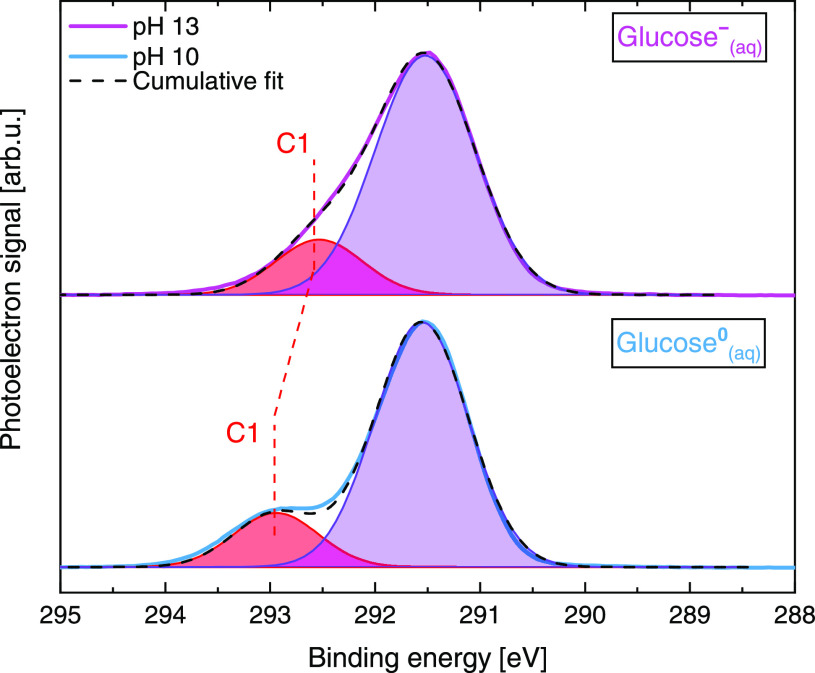
PES spectra of 1 M glucose aqueous solutions at pH 10 and 13, reproduced from Figure 3. The dashed lines indicate cumulative Gaussian fits. Red and purple fills highlight the associated C1 fit component and single-fit component accounting for C2–C6, respectively.

With the aim of identifying the individual spectral contribution from the C1–OH group in the experimental data, we performed a cumulative Gaussian fit analysis of the pH 10 and pH 13 glucose C 1s PES spectra from Figure 3 (glucose^0^_(aq)_ and glucose^–^_(aq)_, respectively). The results are presented in Figure 6.

Both the pH 10 (Figure 6, bottom) and pH 13 (Figure 6, top) spectra were fit using two Gaussian curves with the area ratio between the C1 (higher energy shoulder) and the main C2–C6 feature constrained to 1:5. This is indeed a sensible approach because in the pH 10 spectrum, the C1 contribution (highlighted in red) is sufficiently separated from the signal of all of the other C sites (in agreement with our calculations, which indicate a 1.2 eV peak separation; see Table S2 in the SI). The main feature (highlighted in purple) is composed of five C contributions, that is, a convolution of the C2–C6 site signals. The obtained C1 and C2–C6 component BEs are 292.9 ± 0.2 and 291.5 ± 0.2 eV for glucose^0^_(aq)_ and 292.5 ± 0.2 and 291.5 ± 0.2 eV for glucose^–^_(aq)_. The 0.4 eV C1 energy shift toward lower BEs in going from glucose^0^_(aq)_ to glucose^–^_(aq)_ reflects the larger electron density at the respective carbon site due to the deprotonation of the associated hydroxyl group. As for the valence spectra, the overall error is a combination of the fitting error reported from the least-squares fitting procedure, the experimental resolution, and the error associated with the calibration of the BE scale (calibrated using liquid water’s O 1s core level). Because of the somewhat higher photon energy used, the experimental energy resolution is slightly worse, amounting to 0.28 eV (PETRA III) and to 0.38 eV (BESSY II). However, for the C 1s BE values, the calibration errors for the BEs are less of a concern because one can reasonably expect that the O 1s core level is hardly affected by the solute (at least when non-surface-active solutes like glucose are studied). Furthermore, the relative shift of the C1-associated peak can be determined independent of any energy-scale calibration.

The choice of Gaussian fit functions is justified because the aqueous-phase signals are predominantly subject to inhomogeneous (environmental) broadening, yielding principal Gaussian energy broadening terms. We also performed cumulative Voigt-profile fits to capture possible lifetime broadening in core-level photoionization,^83^ but the results did not converge due to the greater number of degrees of freedom in those fits. However, with an expected 80–100 meV lifetime broadening component of the C 1s peak, which is one order of magnitude smaller than the Gaussian broadening contribution, we do not expect a Voigt fit (either unconstrained or constrained to known lifetime-broadened widths) to yield any appreciable difference in our fit results. In a related context, we have also performed fitting analyses where each C site was represented by a Gaussian with essentially the same peak area and width. Although such a procedure can accurately reproduce the experimental PES spectra, the fits are not unique, and they yield very large fitting errors due to the similar energies of several components. Accordingly, an experimentally meaningful distinction between the individual C2 to C6 BEs is not possible. This explains the use of a single C2–C6 Gaussian in Figure 6, which yields meaningful fitting errors below 100 meV.

Our analysis implies that the C 1s photoionization cross-sections are taken to be identical for the six C sites, which can be considered a reasonable assumption over such a narrow BE range, well above the photoionization threshold. Note that at the near-magic-angle electron collection geometry adopted in the majority of our experiments, the differential photoionization cross-section becomes essentially independent of the β parameter, which could vary with the molecular shape and character at the different C sites.^56^

### Determination of p*K*_a1_ and Associated Deprotonated Structures

**Figure 7:**
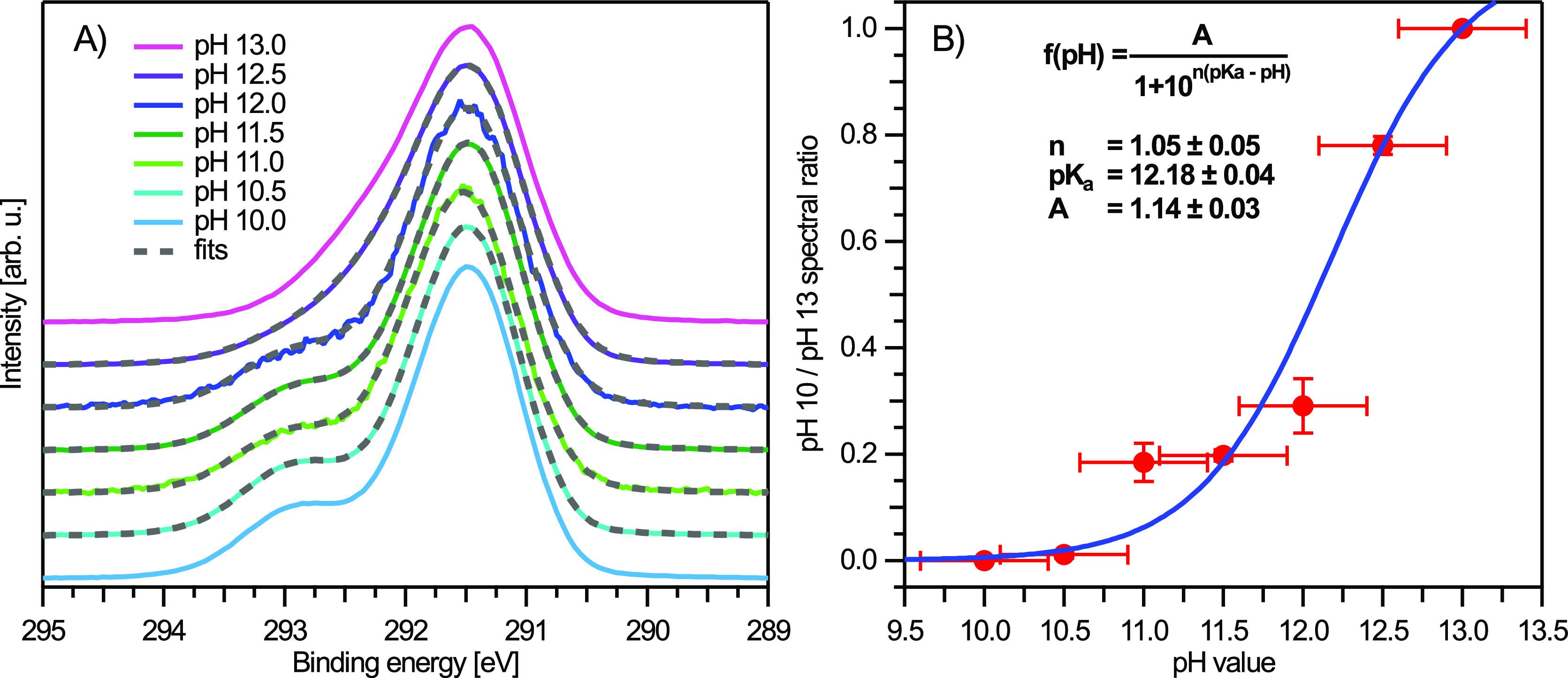
(A) Fits to C 1s PES spectra for intermediate pH values (10.5–12.5) with a model combining the pH 10 (light blue) and pH 13 (magenta) spectra; see the text for details. All spectra were area-normalized and shifted in energy to match the pH 10 spectrum prior to fitting. The vertical offset is added for clarity. (B) Resulting ratio (pH 10/pH 13 spectral contribution) from the fits in panel A as a function of pH value. A fit of a rearranged Henderson–Hasselbalch equation^84^ yields a p*K*_a1_ value of 12.18 ± 0.04, but considering uncertainties in the pH values when preparing the solutions, 12.2 ± 0.2 is deemed a reasonable result.

To determine the p*K*_a1_ value purely from the C 1s PES data, we took the representative experimental spectra of glucose^0^_(aq)_ (pH 10) and glucose^–^_(aq)_ (pH 13) to act as basis curves to fit the spectra associated with the intermediate pH values (10.5–12.5) and determined the ratios of the glucose^0^_(aq)_ versus glucose^–^_(aq)_ spectral contributions. All spectra were area-normalized, and the main C 1s feature was overlapped in energy prior to fitting. The basis curves were fit to each spectrum, with the relative basis curve ratio defined as a fitting parameter using the following relation: Signal_pH 10_*ratio + Signal_pH 13_*(1 – ratio). The results of the fits are shown in Figure 7A, whereas the resulting ratio values are plotted in Figure 7B; the latter can be considered a “photoemission spectroscopy titration”. We also fit the data shown in Figure 7B with a rearranged Henderson–Hasselbalch equation.^84^ The resulting p*K*_a1_ value was found to be 12.18 ± 0.04. Considering additional uncertainties in the pH values when preparing the solutions, a precision of 0.2, yielding 12.2 ± 0.2, is reasonable. This result is in excellent agreement with the value reported from titration-based methods (p*K*_a1_ values in the 12.1 to 12.5 range^36,37,39−41^). The novelty of our LJ-PES approach is the simultaneous determination of the actual deprotonation site, which allows us to associate site-selective spectral changes with a particular acid-ionization constant. Note that our data do not encompass the second acidity constant p*K*_a2_, which is expected at a pH of 13.9.^36,37^ This is also the likely reason that the curve in Figure 7B does not end in a plateau and that rather aqueous-phase glucose is already starting to transition into the double-deprotonated species at higher pH values. Our simple analysis thus does not cover the full picture and is rather meant as a first demonstration of the feasibility of this approach.

To further confirm the assignment of the deprotonation site, we calculated p*K*_a_ values of glucose upon acid ionization at different C–OH groups. Our results show a robust trend: The p*K*_a_ value corresponding to acid ionization at C1 is always 1–3 p*K*_a_ units below the others, irrespective of the method used; the second most easily ionizable C–OH group is located at the C4 site. The calculated values are shown in Table 2, and further analysis is presented in the SI in Table S3. Our results are in agreement with findings by Feng et al.,^85^ who showed that C1 is the best proton donor and is associated with the highest acidity, followed by C4. Notably, though, we observe no significant differences in the acidity constants between the two anomeric forms. This latter finding is in contradiction with the report by Feng et al.,^85^ who found p*K*_a1_ values for C4 and C6 sites to vary between the anomeric forms by more than 2 and 4 p*K*_a_ units, respectively. Whereas charge sharing between the two most acidic (C1 and C4) sites has been suggested by Lewis and Schramm^39^ and cannot be completely excluded based on the C 1s PES experimental data, the present calculations show that C4-deprotonation contributions to the p*K*_a1_ equilibrium would be negligibly small. The second acid dissociation constant (p*K*_a2_) of glucose, assuming deprotonation at C1 followed by deprotonation at C4, was also calculated, yielding a value of 20.8, which is well-separated from the p*K*_a1_ value.

**Table 2:** Glucose Acidity Constants Calculated Using the Thapa and Schlegel^69^ Approach and Assuming a Mixed Explicit/Implicit Model with a Single Solvating Molecule

	C1–OH	C2–OH	C3–OH	C4–OH	C6–OH
calculated p*K*_a1_	11.3	15.4	15.5	14.3	17.3

**Figure 8:**
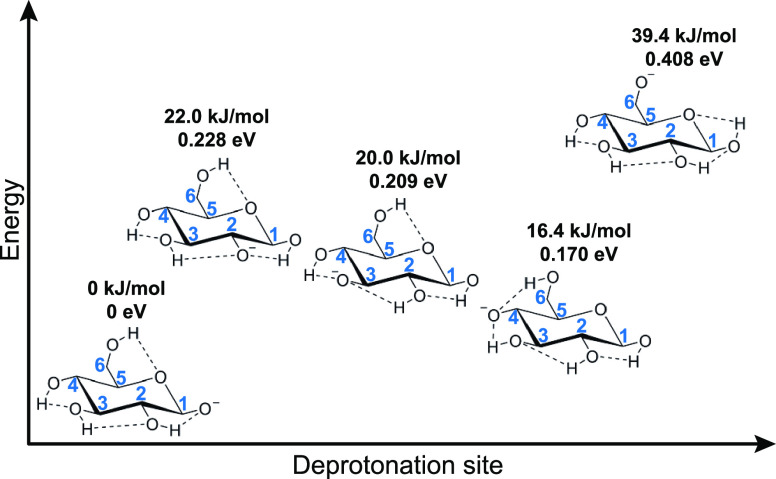
Calculated molecular structures of aqueous-phase β-glucose deprotonated at different C–OH groups. Relative energies (kJ/mol and eV) are also shown and were obtained using free-energy calculations. The presented structures were found as local energetic minima.

The values of the acidity constants are controlled by the energetics of the glucose anion produced during deprotonation, which are strongly influenced by the presence of the hydrogen-bonding network, including both glucose–glucose and glucose–water interactions.^40,46,79,86−88^ A summary of the different calculated deprotonated structures of glucose^–^_(aq)_, together with the respective energies, is shown in Figure 8. Using a relative energy scale, it can be seen that the C1-deprotonated form is energetically preferred.

## Conclusions

We have investigated the pH-dependent molecular structure changes in glucose by performing LJ-PES experiments over the valence and C 1s spectral regions. With the support of electronic structure calculations, we show how aqueous-phase PES data can be applied to determine the first acid dissociation constant (p*K*_a1_) and, more importantly, to unambiguously identify the deprotonation site.

We reported the lowest vertical ionization energies (VIEs), that is, binding energies (BEs) of aqueous-phase glucose from 1 M solutions at pH values below and above p*K*_a1_ as 9.09 and 7.95 eV, respectively. In addition, our calculations confirmed the signatures of protonated (glucose^0^_(aq)_) and deprotonated (glucose^–^_(aq)_) species in the experimental data. An experimental VIE of ∼8.5 eV was determined for glucose^–^_(aq)_, almost 1 eV lower than that for its protonated counterpart, glucose^0^_(aq)_.

We have also reported two C 1s PES fingerprints of aqueous glucose: a main C 1s photoelectron feature with a BE of 291.5 eV and a less intense, higher energy feature at 293 eV. Our calculations show that the latter originates from the photoionization of the C1–OH group in glucose. We found that the C1–OH C 1s BE shifts by 0.4 eV toward lower values upon the deprotonation of glucose, as evident from data recorded at pH values below and above p*K*_a1_ as well as from our calculations. Our combined experimental and theoretical approach confirms that at p*K*_a1_ deprotonation occurs almost exclusively at the C1 site (in contrast, deprotonation at the C4 site is negligibly small) and that a cyclic deprotonated structure prevails.

The sensitivity of LJ-PES to local chemical environments enables us to identify spectral fingerprints of pH-dependent, site-specific deprotonation in glucose. In particular, our studies provide a deeper understanding of the correlation between the molecular structure and the biological function in aqueous-phase glucose as well as in other pyranose-based sugars more generally. Thus we demonstrate the use of solution-phase PES as a general methodology to determine p*K*_a_ values, expanding on the capabilities of the technique to investigate the acid–base chemistry and structure–function relationship of polyprotic acids.

## Supporting Information Available

Calculated valence VIEs for aqueous-phase glucose α- and β-anomers in their protonated (glucose^0^_(aq)_) and deprotonated (glucose^–^_(aq)_) forms. Table S1. Calculated C 1s VIEs for aqueous-phase α- and β-glucose as well as linear (noncyclic, n-) glucose in their protonated (glucose^0^_(aq)_) and deprotonated (glucose^–^_(aq)_) forms. Table S2. First and second acidity constants (p*K*_a1_ and p*K*_a2_, respectively) calculated for different C–OH groups in α- and β-glucose. Table S3. Sample input for calculations of valence VIEs. Cartesian coordinates of the structures used to calculate valence VIEs. Sample input for calculations of C 1s VIEs. Cartesian coordinates of the structures used to calculate C 1s VIEs. Sample input for calculations of p*K*_a_ values. Cartesian coordinates of the structures used to calculate p*K*_a_ values. Sample inputs for calculations of p*K*_a_ values via the thermodynamic cycle. Cartesian coordinates of structures used to calculate p*K*_a_ values via a thermodynamic cycle (PDF)

## Notes

Data relevant for this study have been deposited at 10.5281/zenodo.4777079.

## Footnotes

a This is the commonly applied procedure in LJ-PES experiments where solute and solvent energies from aqueous solutions are determined from the energy difference between a solute/solvent peak position and the well-known energies of simultaneously measured water gas-phase 1b_1_ or 1a_1_ (O 1s) peak features. More precisely, the gas-phase referenced ionization energy of liquid water 1b_1(l)_ (or O 1s_(l)_) has been used as an internal aqueous-solution reference, although the reference was only determined for neat liquid water. As discussed in ref (89), this gas-phase referencing method generally does not produce accurate absolute liquid-phase BEs because of the typically ill-defined charging of the solution surface. Accurate BEs can, however, be obtained when measuring the respective photoelectron peak along with the so-called low-energy spectral cutoff. Note, though, that accurately determined 1b_1_ and O 1s BEs from liquid water would still not serve as useful references to infer solute energies, as they neglect any solute-induced effects on the electronic structure of neat liquid water. Despite this, C 1s BEs reported in the present work are expected to be accurate within 200–500 meV of the true values.
