# Supplementary material for: Following in Emil Fischer’s Footsteps: A Site-Selective Probe of Glucose Acid–Base Chemistry
Source: J Phys Chem A. 2021 Jul 30;125(32):6881–92. doi: 10.1021/acs.jpca.1c04695 (PMC8381351; doi:10.1021/acs.jpca.1c04695)
Supplement: Supplementary file 1 — jp1c04695_si_001.pdf [file jp1c04695_si_001.pdf]

Supporting Information

Following in Emil Fischer's Footsteps: a Site-Selective Probe of Glucose Acid-Base Chemistry

Sebastian Malerz,[1,#] Karen Mudryk,[1,#] Lukáš Tomaník,[2] Dominik Stemer,[1] Uwe Hergenhahn,[1] Tillmann Buttersack,[1] Florian Trinter,[1,3] Robert Seidel,[4,5] Wilson Quevedo,[4] Claudia Goy,[6] Iain Wilkinson,[7] Stephan Thürmer,[8] Petr Slavíček,[2,] and Bernd Winter[1,*]*

[1] Fritz-Haber-Institut der Max-Planck-Gesellschaft, Faradayweg 4-6, 14195 Berlin, Germany

[2] Department of Physical Chemistry, University of Chemistry and Technology, Technická 5, Prague 6, 16628, Czech Republic

[3] Institut für Kernphysik, Goethe-Universität, Max-von-Laue-Straße 1, 60438 Frankfurt am Main, Germany

[4] Operando Interfacial Photochemistry, Helmholtz-Zentrum Berlin für Materialien und Energie, Albert-Einstein-Straße 15, 12489 Berlin, Germany

[5] Humboldt-Universität zu Berlin, Institut für Chemie, Brook-Taylor-Str. 2, 12489 Berlin, Germany

[6] Centre for Molecular Water Science (CMWS), Photon Science, Deutsches Elektronen-Synchrotron (DESY), Notkestraße 85, 22607 Hamburg, Germany

[7] Department of Locally-Sensitive & Time-Resolved Spectroscopy, Helmholtz-Zentrum Berlin für Materialien und Energie, Hahn-Meitner-Platz 1, 14109 Berlin, Germany

[8] Department of Chemistry, Graduate School of Science, Kyoto University, Kitashirakawa-Oiwakecho, Sakyo-Ku, Kyoto 606-8502, Japan

# Authors contributed equally

* Corresponding authors: petr.slavicek@vscht.cz; winter@fhi-berlin.mpg.de

Table S1. Calculated valence vertical ionization energies (VIEs) for aqueous-phase glucose α- and β- anomers in their protonated (glucose$^{0}_{(aq)}$) and deprotonated (glucose$^{-}_{(aq)}$) forms. For glucose$^{-}_{(aq)}$, VIEs are shown for deprotonation taking place at different C–OH groups. Results obtained from the inclusion of one, two, or six explicit water molecules (w) in our calculations are also presented. Gas-phase VIEs calculated for β-D-glucose in the glucose$^{0}_{(aq)}$ and glucose$^{-}_{(aq)}$ (deprotonated at C1) forms are shown for comparison. All values are in eV.

	**Aqueous-phase α-D-glucose**		
**Glucose structure**	**α-D-glucose$^{0}_{(aq)}$**	**α-D-glucose$^{-}_{(aq)}$ (C1-O^{-})**	
**1. VIE**	9.06	6.81	
**2. VIE**	9.61	7.63	
**3. VIE**	9.71	8.82	
	**Gas-phase β-D-glucose**		
**Glucose structure**	**β-D-glucose$^{0}_{(g)}$**	**β-D-glucose$^{-}_{(g)}$ (C1-O^{-})**	
**1. VIE**	9.99	3.56	
**2. VIE**	10.23	4.47	
**3. VIE**	10.38	5.70	
	**Aqueous-phase β-D-glucose$^{0}_{(aq)}$**		
**Glucose structure**	**β-D-glucose$^{0}_{(aq)}$**	**β-D-glucose$^{0}_{(aq)}$ (1w)**	**β-D-glucose$^{0}_{(aq)}$ (2w)**
**1. VIE**	9.11	9.00	9.09
**2. VIE**	9.44	9.42	9.40
**3. VIE**	9.67	9.67	9.66
	**Aqueous-phase β-D-glucose$^{-}_{(aq)}$ (deprotonation at C1)**		
**Glucose structure**	**β-D-glucose$^{-}_{(aq)}$ (C1-O^{-})**	**β-D-glucose$^{-}_{(aq)}$ (C1-O^{-}, 1w)**	**β-D-glucose$^{-}_{(aq)}$ (C1-O^{-}, 6w)**
**1. VIE**	6.75	7.16	7.95
**2. VIE**	7.65	7.97	8.87
**3. VIE**	9.00	9.04	9.44
	**Aqueous-phase β-D-glucose$^{-}_{(aq)}$ (deprotonation at C2)**		
**Glucose structure**	**β-D-glucose$^{-}_{(aq)}$ (C2-O^{-})**	**β-D-glucose$^{-}_{(aq)}$ (C2-O^{-}, 1w)**	**β-D-glucose$^{-}_{(aq)}$ (C2-O^{-}, 6w)**
**1. VIE**	6.74	7.28	8.09
**2. VIE**	7.10	7.42	8.32
**3. VIE**	9.31	9.30	9.31
	**Aqueous-phase β-D-glucose$^{-}_{(aq)}$ (deprotonation at C3)**		
**Glucose structure**	**β-D-glucose$^{-}_{(aq)}$ (C3-O^{-}, 1w)**	**β-D-glucose$^{-}_{(aq)}$ (C3-O^{-})**	**β-D-glucose$^{-}_{(aq)}$ (C3-O^{-}, 6w)**
**1. VIE**	7.26	6.69	7.71
**2. VIE**	7.58	7.15	7.79
**3. VIE**	9.24	9.33	8.90
	**Aqueous-phase β-D-glucose$^{-}_{(aq)}$ (deprotonation at C4)**		
**Glucose structure**	**β-D-glucose$^{-}_{(aq)}$ (C4-O^{-}, 6w)**	**β-D-glucose$^{-}_{(aq)}$ (C4-O^{-}, 1w)**	**β-D-glucose$^{-}_{(aq)}$ (C4-O^{-})**
**1. VIE**	7.99	7.13	6.96
**2. VIE**	8.33	7.38	7.20
**3. VIE**	8.98	8.65	8.85
	**Aqueous-phase β-D-glucose$^{-}_{(aq)}$ (deprotonation at C6)**		
**Glucose structure**	**β-D-glucose$^{-}_{(aq)}$ (C6-O^{-})**	**β-D-glucose$^{-}_{(aq)}$ (C6-O^{-}, 1w)**	**β-D-glucose$^{-}_{(aq)}$ (C6-O^{-}, 6w)**
**1. VIE**	6.17	6.79	7.92
**2. VIE**	6.45	6.91	8.01
**3. VIE**	9.33	8.97	9.30

The sample input and cartesian coordinates of the structures used to calculate the values presented in this table are shown in pages 5 and 6-9, respectively.

Table S2. Calculated C 1s vertical ionization energies (VIEs) for aqueous-phase α- and β-glucose as well as linear (non-cyclic, n-) glucose in their protonated (glucose$^{0}_{(aq)}$) and deprotonated (glucose$^{-}_{(aq)}$) forms. For glucose$^{-}_{(aq)}$, VIEs are shown for deprotonation taking place at different C–OH groups. Results obtained from the inclusion of one explicit water molecule (w) in our calculations are also presented. Gas-phase VIEs calculated for β-D-glucose in the glucose$^{0}_{(aq)}$ and glucose$^{-}_{(aq)}$ (deprotonated at C1) forms are shown for comparison. All values are in eV.

			**Aqueous-phase α-D-glucose**			
	**α-D-glucose$^{0}_{(aq)}$**	**α-D-glucose$^{-}_{(aq)}$ (C1-O^{-})**	**α-D-glucose$^{-}_{(aq)}$ (C2-O^{-})**	**α-D-glucose$^{-}_{(aq)}$ (C3-O^{-})**	**α-D-glucose$^{-}_{(aq)}$ (C4-O^{-})**	**α-D-glucose$^{-}_{(aq)}$ (C6-O^{-})**
C1	292.82	291.15	291.66	292.32	292.46	292.48
C2	291.27	290.16	289.77	290.25	290.87	291.04
C3	291.13	290.72	290.22	289.59	290.42	290.87
C4	291.14	290.67	290.90	290.13	289.79	290.64
C5	291.17	290.51	290.84	290.71	290.27	290.18
C6	291.09	290.56	290.72	290.86	290.22	289.46
			**Aqueous-phase β-D-glucose**			
	**β-D-glucose$^{0}_{(aq)}$**	**β-D-glucose$^{-}_{(aq)}$ (C1-O^{-})**	**β-D-glucose$^{-}_{(aq)}$ (C2-O^{-})**	**β-D-glucose$^{-}_{(aq)}$ (C3-O^{-})**	**β-D-glucose$^{-}_{(aq)}$ (C4-O^{-})**	**β-D-glucose$^{-}_{(aq)}$ (C6-O^{-})**
C1	292.77	291.17	291.56	292.21	292.33	292.44
C2	291.26	290.17	289.55	290.11	290.75	291.04
C3	291.26	290.65	290.06	289.55	290.18	291.00
C4	291.17	290.77	290.85	290.00	289.70	290.68
C5	291.25	290.77	290.85	290.69	290.27	290.27
C6	291.12	290.76	290.61	290.80	290.36	289.49
			**Aqueous-phase n-D-glucose**			
	**n-D-glucose$^{0}_{(aq)}$**		**n-D-glucose$^{-}_{(aq)}$ (C2-O^{-})**	**n-D-glucose$^{-}_{(aq)}$ (C3-O^{-})**	**n-D-glucose$^{-}_{(aq)}$ (C4-O^{-})**	**n-D-glucose$^{-}_{(aq)}$ (C6-O^{-})**
C1	292.84		291.89	292.07	291.80	292.51
C2	291.61		290.11	290.39	290.99	291.33
C3	291.46		290.34	290.06	290.17	291.10
C4	291.37		290.69	290.37	289.87	290.77
C5	291.36		290.99	290.74	290.61	290.05
C6	291.12		290.90	290.77	290.41	289.59
			**Aqueous-phase α- and β-D-glucose with one explicit water molecule**			
		**α-D-glucose$^{0}_{(aq)}$ (1w)**	**α-D-glucose$^{-}_{(aq)}$ (C1-O^{-}, 1w)**	**β-D-glucose$^{0}_{(aq)}$ (1w)**	**β-D-glucose$^{-}_{(aq)}$ (C1-O^{-}, 1w)**	
C1		292.59	291.36	292.56	291.37	
C2		291.15	290.34	291.14	290.31	
C3		291.06	290.64	291.21	290.83	
C4		291.09	290.74	291.13	290.83	
C5		291.09	290.61	291.18	290.75	
C6		291.05	290.78	291.08	290.83	
			**Gas-phase phase α- and β-D-glucose**			
		**α-D-glucose$^{0}_{(g)}$**	**α-D-glucose$^{-}_{(g)}$ (C1-O^{-})**	**β-D-glucose$^{0}_{(g)}$**	**β-D-glucose$^{-}_{(g)}$ (C1-O^{-})**	
C1		293.97	288.02	293.86	287.98	
C2		292.30	287.22	292.26	287.19	
C3		292.02	287.71	292.17	288.04	
C4		291.96	287.99	292.00	288.06	
C5		292.07	287.60	292.17	287.83	
C6		291.98	288.10	291.99	288.19	

The sample input and cartesian coordinates of the structures used to calculate the values presented in this table are shown in pages 10-11 and 12-15, respectively.

Table S3. First and second acidity constants (pK_{a1} and pK_{a2} , respectively) caclulated for different C–OH groups in α- and β-glucose. The results were obtained using two different approaches (according to Thapa and Schlegel [1] and based on a thermodynamic cycle, see Methods in the main text for details) and from upgraded calculations involving one explicit water molecule (1w). The pK_{a2} calculations consider deprotonation at C4 after previous deprotonation at C1 (pK_{a1}). PT stands for 'proton transfer' (proton transfer from C4 to C6 occurs during geometry optimization and the respective value cannot be determined).

		**α-D-glucose$^0_{(aq)}$ Schlegel**	**β-D-glucose$^0_{(aq)}$ Schlegel**	**β-D-glucose$^0_{(aq)}$ Schlegel, 1w**	**β-D-glucose$^0_{(aq)}$ Schlegel, 2w**	**α-D-glucose$^0_{(aq)}$ thermodynamic cycle**	**β-D-glucose$^0_{(aq)}$ thermodynamic cycle**	**β-D-glucose$^0_{(aq)}$ thermodynamic cycle, 1w**
	C1-OH	16.2	16.1	13.5	11.3	18.6	18.9	17.6
	C2-OH	19.4	20.0	16.9	15.4	21.3	22.4	
pK_{a1}	C3-OH	20.3	19.7	17.1	15.5	23.1	22.0	
	C4-OH	18.8	19.0	16.4	14.3	19.1	19.8	19.4
	C6-OH	22.8	23.0	19.6	17.3	PT	20.9	
pK_{a2}		20.4	19.9	19.9	20.8	21.3	20.8	20.2

The sample input and cartesian coordinates of the structures used to calculate the values presented in this table are respectively shown in pages 16 and 17-20 (Schlegel) and 21 and 22-24 (thermodynamic cycle).

Sample input for calculations of valence vertical ionization energies using Gaussian 09 (revision D.01)

```
%Mem=8GB
%NProcShared=8
%chk=IE-C1-1
#p CAM-B3LYP/6-31+g* nosymm scrf(pcm,solvent=water,read)

comment

-1 1
 C   -0.336686    0.306387    0.038697
 C   -0.101301   -0.459861    1.344344
 O    1.204342   -0.210132    1.836482
 C    1.458081    1.218691    2.162071
 C    1.317507    1.967493    0.824981
 C   -0.061302    1.787168    0.236451
 C   -0.212906   -1.967276    1.178653
 O    0.837890   -2.481110    0.368347
 O    2.636654    1.409859    2.721777
 O    1.567992    3.350702    1.044804
 O   -0.215811    2.413051   -1.039066
 O   -1.682860    0.090436   -0.370853
 H    2.070978    1.561994    0.133773
 H    2.281515    3.348743    1.712723
 H   -0.808952    2.199752    0.929046
 H   -0.069834    3.365277   -0.928924
 H    0.348599   -0.079288   -0.727786
 H   -1.859657    0.692881   -1.111192
 H   -0.858548   -0.134424    2.076544
 H   -1.153222   -2.230975    0.690954
 H   -0.186967   -2.434278    2.171662
 H    1.658341   -2.078330    0.699507
 H    0.594450    1.499069    2.814648

noneq=write

--link1--
%NprocShared=8
%mem=8GB
%chk=IE-C1-1
# CAM-B3LYP/6-31+g* nosymm Geom=check Guess=Read scrf(pisalr,pcm,solvent=water,read) td(nstates=2,root=1,noneq) IOP(10/74=10)

comment

0 2

NonEq=Read
```

Cartesian coordinates of the structures used to calculate valence vertical ionization energies. Optimization was performed on the CAM-B3LYP/6-31+G* level of theory using the polarizable continuum model (PCM). Minima were confirmed by the absence of imaginary vibrational frequencies.

α-D-glucose$^{0}_{(aq)}$

O	-0.277514	0.355476	0.022786
C	-0.129614	-0.276824	1.272020
C	1.295479	-0.118901	1.802972
C	1.703862	1.350083	1.797730
C	1.497975	1.951694	0.418994
C	0.050373	1.750895	-0.021107
H	-0.366543	-1.327712	1.090249
O	-0.981027	0.264515	2.253106
H	-1.889959	-0.038969	2.105806
H	1.973863	-0.678156	1.153309
O	1.428675	-0.682781	3.096546
H	0.663833	-0.390128	3.621846
H	1.089558	1.904215	2.520409
O	3.078415	1.507178	2.123890
H	3.222746	1.157752	3.017466
H	2.164550	1.448131	-0.293457
O	1.760318	3.346928	0.426033
H	2.634569	3.478987	0.826850
H	-0.611844	2.309850	0.651523
C	-0.221358	2.191262	-1.448168
H	0.051642	3.241221	-1.564578
H	-1.293231	2.081496	-1.653964
O	0.544590	1.458953	-2.393979
H	0.346387	0.517493	-2.266946

α-D-glucose$^{-}_{(aq)}$ (C1-O^{-})

C	-0.279301	0.347442	0.022742
O	-0.130180	-0.327757	1.260655
C	1.196266	-0.199759	1.910063
C	2.241034	-0.765990	0.916213
C	2.099696	-0.119463	-0.453673
C	0.691277	-0.252397	-0.992073
O	1.526642	1.026963	2.293812
O	3.534090	-0.473317	1.440310
O	2.983824	-0.705864	-1.412476
O	0.553185	0.439841	-2.230029
C	-1.739956	0.190657	-0.367182
O	-2.138512	-1.176198	-0.356405
H	1.067790	-0.920663	2.743498
H	2.156150	-1.853747	0.819639
H	3.359782	0.365357	1.926811
H	2.333347	0.949107	-0.361025
H	3.887714	-0.609670	-1.073043
H	0.452236	-1.315090	-1.135212
H	1.315557	0.196968	-2.779944
H	-0.058489	1.418188	0.134568
H	-1.909393	0.567811	-1.377670
H	-2.357399	0.768136	0.333081
H	-1.781105	-1.552823	0.465724

β-D-glucose$^{0}_{(aq)}$

C	-0.323580	0.297702	0.037777
O	-0.139344	-0.418822	1.259449
C	1.128215	-0.209226	1.860758
C	2.227584	-0.713367	0.933357
C	2.115364	-0.020196	-0.413320
C	0.713666	-0.169673	-0.982729
O	3.516330	-0.427316	1.448124
O	3.013987	-0.565542	-1.368363
O	0.558655	0.615911	-2.153877
C	-1.757614	0.042869	-0.392120
O	-2.027106	-1.339480	-0.573435
H	2.095261	-1.795840	0.804854
H	3.642797	-0.933455	2.265829
H	2.329642	1.049819	-0.278097
H	3.921039	-0.417825	-1.057947
H	0.534074	-1.227838	-1.212124
H	1.281871	0.387233	-2.759743
H	-0.197850	1.376769	0.212513
H	-1.944274	0.530010	-1.350445
H	-2.432499	0.471778	0.358658
H	-1.819074	-1.792944	0.258928
O	1.192518	-0.950797	3.034386
H	0.685581	-0.507215	3.731942
H	1.256527	0.864170	2.064609

β-D-glucose$^{0}_{(aq)}$ (1w)

C	0.748399	-0.313649	-1.207166
C	-0.309036	0.237966	-0.250897
O	-0.146359	-0.359370	1.034350
C	1.114079	-0.088098	1.640353
C	2.225293	-0.671662	0.775149
C	2.138602	-0.103803	-0.629319
C	-1.733756	-0.065487	-0.681213
O	-1.994814	-1.461136	-0.720665
O	1.160783	-0.699478	2.878110
O	3.505380	-0.343103	1.287995
O	3.057931	-0.726129	-1.516600
O	0.610524	0.361235	-2.448060
H	2.093262	-1.761618	0.742907
H	3.579406	-0.719284	2.179485
H	2.344553	0.975550	-0.587588
H	3.958045	-0.548777	-1.201670
H	0.578262	-1.389379	-1.343266
H	1.349220	0.087466	-3.015339
H	-0.191153	1.329731	-0.176676
H	-1.904116	0.317525	-1.688607
H	-2.424669	0.435268	0.008165
H	-1.779379	-1.824043	0.153445
H	0.713317	-0.133424	3.549067
H	1.231661	1.003121	1.731501
O	-0.034238	0.878630	4.772606
H	-0.962964	1.100703	4.603315
H	0.411502	1.725481	4.929800

β-D-glucose$^{0}_{(aq)}$ (2w)

C	2.215750	0.014615	-0.610984
C	0.826814	-0.322680	-1.129934
C	-0.241206	0.208857	-0.175718
O	0.010393	-0.295223	1.140765
C	1.270535	0.089013	1.691515
C	2.388740	-0.466053	0.817907
O	0.609373	0.268208	-2.401746
C	-1.658426	-0.183420	-0.569613
O	-1.844923	-1.576209	-0.737890
O	1.380941	-0.463175	2.951939
O	3.658799	-0.023040	1.264056
O	3.147952	-0.592984	-1.494717
O	0.171211	1.139793	4.805526
H	2.331731	-1.562461	0.849019
H	3.804311	-0.366822	2.159715
H	2.346824	1.106117	-0.635245
H	4.042963	-0.337393	-1.221828
H	0.726564	-1.413100	-1.199686
H	1.353078	0.016296	-2.972659
H	-0.190817	1.307993	-0.154243
H	-1.888485	0.288031	-1.527389
H	-2.349063	0.216855	0.183777
H	-1.830060	-2.007797	0.143050
H	0.930530	0.113473	3.613009
H	1.315589	1.187910	1.728015
H	-0.761840	1.341620	4.634238
H	0.603007	1.997581	4.941083
H	-0.814934	-1.793417	1.908594
O	-1.531004	-2.461974	1.900753
H	-1.101104	-3.322410	2.016381

β-D-glucose$^{-}_{(aq)}$ (C1-O^{-})

C	-0.336686	0.306387	0.038697
C	-0.101301	-0.459861	1.344344
O	1.204342	-0.210132	1.836482
C	1.458081	1.218691	2.162071
C	1.317507	1.967493	0.824981
C	-0.061302	1.787168	0.236451
C	-0.212906	-1.967276	1.178653
O	0.837890	-2.481110	0.368347
O	2.636654	1.409859	2.721777
O	1.567992	3.350702	1.044804
O	-0.215811	2.413051	-1.039066
O	-1.682860	0.090436	-0.370853
H	2.070978	1.561994	0.133773
H	2.281515	3.348743	1.712723
H	-0.808952	2.199752	0.929046
H	-0.069834	3.365277	-0.928924
H	0.348599	-0.079288	-0.727786
H	-1.859657	0.692881	-1.111192
H	-0.858548	-0.134424	2.076544
H	-1.153222	-2.230975	0.690954
H	-0.186967	-2.434278	2.171662
H	1.658341	-2.078330	0.699507
H	0.594450	1.499069	2.814648

β-D-glucose$^{-}_{(aq)}$ (C2-O^{-})

C	-0.336686	0.306387	0.038697
C	-0.101301	-0.459861	1.344344
O	1.204342	-0.210132	1.836482
C	1.458081	1.218691	2.162071
C	1.317507	1.967493	0.824981

β-D-glucose$^{-}_{(aq)}$ (C3-O^{-})

C	-0.263466	0.307246	-0.003324
C	-0.157568	-0.431364	1.325820
O	1.091891	-0.103007	1.952009
C	1.329558	1.281762	2.204383
C	1.256802	2.036931	0.887146

β-D-glucose$^{-}_{(aq)}$ (C4-O^{-})

C	-0.317025	0.282643	-0.005442
C	-0.112191	-0.431784	1.341963
O	1.212934	-0.185362	1.833796
C	1.457525	1.177014	2.063500
C	1.324658	1.988572	0.774432

C	-0.061302	1.787168	0.236451
C	-0.212906	-1.967276	1.178653
O	0.837890	-2.481110	0.368347
O	2.636654	1.409859	2.721777
O	1.567992	3.350702	1.044804
O	-0.215811	2.413051	-1.039066
O	-1.682860	0.090436	-0.370853
H	2.070978	1.561994	0.133773
H	2.281515	3.348743	1.712723
H	-0.808952	2.199752	0.929046
H	-0.069834	3.365277	-0.928924
H	0.348599	-0.079288	-0.727786
H	-1.859657	0.692881	-1.111192
H	-0.858548	-0.134424	2.076544
H	-1.153222	-2.230975	0.690954
H	-0.186967	-2.434278	2.171662
H	1.658341	-2.078330	0.699507
H	0.594450	1.499069	2.814648

C	-0.108168	1.808074	0.231403
C	-0.172197	-1.945657	1.217982
O	0.940713	-2.444072	0.486353
O	2.559256	1.381267	2.853588
O	1.430864	3.431103	1.072625
O	-0.281621	2.561921	-0.895476
O	-1.529527	0.070295	-0.606343
H	2.050962	1.648944	0.226902
H	0.926638	3.801458	0.315791
H	-0.865685	2.070253	1.011296
H	0.532737	-0.043276	-0.675586
H	-1.663228	0.872579	-1.151221
H	-0.990089	-0.122335	1.975631
H	-1.070909	-2.266006	0.686891
H	-0.187601	-2.379127	2.226321
H	1.738163	-2.050177	0.875571
H	3.267572	1.186331	2.218089
H	0.583914	1.660137	2.913893

C	-0.062550	1.775130	0.211732
C	-0.288496	-1.935869	1.193131
H	0.495381	-2.320730	0.522109
O	2.759618	1.248169	2.580725
O	1.599040	3.340941	1.133168
O	-0.219225	2.462660	-1.022213
O	-1.556573	0.066221	-0.570494
H	2.079043	1.633245	0.060443
H	1.556974	3.887627	0.333590
H	-0.802992	2.159118	0.931519
H	-0.951046	1.975794	-1.452878
H	0.503181	-0.083147	-0.664030
H	-1.776692	-1.430994	0.064918
H	-0.845960	-0.054643	2.071753
O	-1.580385	-2.212523	0.677309
H	-0.177769	-2.437079	2.161079
H	3.047629	2.173929	2.508902
H	0.737246	1.557665	2.807493

β-D-glucose$^{-}_{(aq)}$ (C6-O^{-})

C	-0.303064	0.263094	0.048407
C	-0.097465	-0.519770	1.343294
O	1.190475	-0.180471	1.883766
C	1.330478	1.182273	2.208498
C	1.229947	2.027354	0.945618
C	-0.099724	1.752933	0.271463
C	-0.164835	-2.048406	1.162280
O	0.664125	-2.578489	0.218486
O	2.599157	1.393195	2.752229
O	1.284658	3.416663	1.230878
O	-0.193762	2.390252	-0.998573
O	-1.624748	0.023644	-0.424769
H	2.052864	1.740674	0.277297
H	2.148362	3.614081	1.625777
H	-0.904495	2.124636	0.922405
H	-0.133731	3.349086	-0.865629
H	0.425846	-0.096316	-0.687646
H	-1.769032	0.613841	-1.181642
H	-0.872229	-0.219321	2.069156
H	-1.244464	-2.251817	0.969336
H	0.018438	-2.447007	2.190098
H	2.626442	1.041076	3.655152
H	0.548443	1.474550	2.927316

β-D-glucose$^{-}_{(aq)}$ (C1-O^{-}, 1w)

C	-0.069965	1.794637	0.217268
C	-0.292086	0.304546	0.021081
C	-0.133443	-0.437698	1.351392
O	1.126353	-0.142490	1.934151
C	1.315476	1.282699	2.247950
C	1.257666	2.022161	0.901779
O	-1.598856	0.047517	-0.479649
C	-0.189490	-1.949464	1.202973
O	0.918251	-2.444633	0.460429
O	2.456903	1.502433	2.894897
O	1.441022	3.415112	1.123532
O	-0.146565	2.399823	-1.073684
H	2.071511	1.637573	0.270213
H	2.121906	3.460681	1.821015
H	-0.875639	2.195363	0.849231
H	-0.049685	3.358655	-0.966608
H	0.456806	-0.072367	-0.687909
H	-1.736527	0.632101	-1.242362
H	-0.949140	-0.124386	2.023074
H	-1.092716	-2.246295	0.666911
H	-0.207724	-2.401343	2.202977
H	1.712552	-2.035056	0.841955
H	0.419822	1.565264	2.846152
H	3.838342	1.067573	2.196434
O	4.747402	0.833025	1.812813
H	4.925435	-0.075333	2.094244

β-D-glucose$^{-}_{(aq)}$ (C2-O^{-}, 1w)

C	1.027527	2.134285	1.373056
C	-0.351669	1.896207	0.764442
C	-0.430171	0.503530	0.169567
C	-0.063473	-0.536816	1.236642
O	1.200005	-0.233052	1.829514
C	1.231903	1.050821	2.429626
O	-0.615713	2.864500	-0.243642
O	-1.743781	0.191777	-0.288263
C	0.063854	-1.947561	0.689297
O	1.119335	-2.060852	-0.255829
O	2.484417	1.196425	3.034212
O	1.181462	3.400920	1.881273
H	1.776947	1.917715	0.580385
H	-1.114052	1.988759	1.554461
H	-0.230303	3.688993	0.108940
H	0.278478	0.428546	-0.664778
H	-2.024953	0.905556	-0.882025
H	-0.846921	-0.532900	2.009990
H	-0.855721	-2.229230	0.173893
H	0.225046	-2.638461	1.526314
H	1.922738	-1.715218	0.165575
H	2.660036	2.156976	2.980851
H	0.439453	1.094674	3.196202
H	2.322307	4.214492	1.202847
O	3.084549	4.756363	0.785458
H	2.714322	5.169430	-0.007129

β-D-glucose$^{-}_{(aq)}$ (C3-O^{-}, 1w)

C	1.002917	1.953855	0.306546
C	-0.348436	1.427844	-0.200833
C	-0.377872	-0.090027	-0.016108
C	-0.151075	-0.465691	1.437777
O	1.117812	0.062969	1.837514
C	1.240447	1.477189	1.741828
O	-0.646348	1.719836	-1.519971
O	-1.633820	-0.570539	-0.473767
C	-0.086032	-1.957721	1.711289
O	0.991453	-2.585047	1.027788
O	2.497831	1.817580	2.235968
O	1.073339	3.369359	0.329060
O	-0.165344	4.113744	-2.023148
H	1.803708	1.544065	-0.332779
H	0.691668	3.729825	-0.506565
H	-1.117959	1.849691	0.483484
H	0.425806	-0.533820	-0.620841
H	-1.826243	0.028042	-1.225402
H	-0.949211	-0.034098	2.061083
H	-1.003837	-2.434491	1.361622
H	0.003969	-2.120431	2.793027

β-D-glucose$^{-}_{(aq)}$ (C4-O^{-}, 1w)

C	0.280938	1.688994	-0.164924
C	-0.009892	0.185834	-0.219381
C	-0.075189	-0.348810	1.228623
O	1.109400	-0.036738	1.967648
C	1.302857	1.355906	2.098986
C	1.479732	1.983053	0.723906
O	-1.171832	-0.135793	-0.903315
C	-0.226529	-1.865475	1.250428
O	-1.396469	-2.260805	0.555073
O	2.475342	1.583631	2.820126
O	1.607041	3.397361	0.808950
O	0.525111	2.178298	-1.480192
H	0.671133	-2.314493	0.797114
H	2.382356	1.549997	0.271943
H	2.417732	3.604348	1.299476
H	-0.599406	2.200563	0.252628
H	0.801692	3.104765	-1.394755
H	0.876028	-0.286716	-0.691857
H	-1.495202	-1.561207	-0.165928
H	-0.945235	0.104734	1.729999
H	-0.291828	-2.219688	2.285070

β-D-glucose$^{-}_{(aq)}$ (C6-O^{-}, 1w)

C	-0.098962	1.735184	0.290140
C	-0.343771	0.255974	0.040408
C	-0.121879	-0.557926	1.313123
O	1.187743	-0.262402	1.826323
C	1.381012	1.091786	2.169033
C	1.259604	1.961516	0.924804
O	-1.682297	0.051921	-0.397495
C	-0.218512	-2.075820	1.094869
O	0.604011	-2.580093	0.115773
O	2.675313	1.247886	2.666082
O	1.360270	3.342634	1.233239
O	-0.218162	2.402018	-0.961430
H	2.051511	1.666013	0.223716
H	2.247421	3.514912	1.585631
H	-0.872756	2.110275	0.975811
H	-0.126766	3.355766	-0.810553
H	0.357403	-0.103934	-0.722038
H	-1.837010	0.655979	-1.141304
H	-0.871038	-0.260471	2.065385
H	-1.289387	-2.273838	0.891354
H	-0.019299	-2.519558	2.095775

H	1.797237	-2.088352	1.243162
H	3.173543	1.548904	1.592247
H	0.514865	1.950890	2.414751
H	-0.458097	3.107459	-1.869110
H	-0.945219	4.666288	-1.869372

H	2.320455	1.399191	3.759366
H	0.437311	1.797355	2.616725
H	-2.460233	0.746697	-0.646989
O	-3.330527	1.260883	-0.512923
H	-3.286202	2.014378	-1.118441

H	2.721505	0.897149	3.568887
H	0.635919	1.392649	2.921925
H	2.044574	-2.528357	0.588035
O	3.018664	-2.438150	0.943170
H	2.968360	-1.596075	1.421319

β-D-glucose$^{-}_{(aq)}$ (C1-O^{-}, 6w)

C	-0.155333	-0.037647	1.712162
O	1.230391	0.128194	2.015529
C	1.688806	1.522800	2.062504
C	1.461418	2.106000	0.658314
C	0.004676	1.977614	0.256869
C	-0.449711	0.534477	0.326121
O	2.983679	1.555849	2.454283
O	1.774860	3.487211	0.590704
O	-0.217023	2.435495	-1.071642
O	-1.848290	0.425720	0.098369
C	-0.468079	-1.521303	1.812543
O	0.291765	-2.305290	0.903407
O	2.657250	-1.710014	3.542142
O	3.758456	0.520163	4.817760
O	4.487405	3.200300	0.993294
O	4.433248	-0.640437	1.520003
H	2.088274	1.539468	-0.045155
H	2.753064	3.579792	0.601478
H	-0.605904	2.574535	0.950888
H	0.169145	3.324326	-1.137177
H	0.097792	-0.049444	-0.424613
H	-2.048572	0.892961	-0.728428
H	-0.765808	0.494891	2.456866
H	-1.517489	-1.685149	1.563225
H	-0.305888	-1.857191	2.844797
H	1.233682	-2.127569	1.054657
H	1.022278	2.034096	2.782694
H	3.917243	0.163550	1.769727
H	4.070065	-1.324795	2.108583
H	3.471526	0.933880	3.956280
H	3.467958	-0.407024	4.714613
H	4.064655	2.495261	1.560356
H	4.980421	2.739670	0.298482
H	2.036207	-1.072919	3.119394
H	2.123782	-2.413224	3.941783
H	2.994796	3.087065	3.545986
O	2.953646	3.841036	4.174494
H	2.878894	3.429180	5.061108
H	3.475635	2.544366	7.229432
O	2.805176	2.321779	6.567108
H	3.165037	1.551022	6.054820

β-D-glucose$^{-}_{(aq)}$ (C2-O^{-}, 6w)

C	1.586216	1.071879	2.451899
C	1.494660	2.213393	1.422677
C	0.122002	2.086033	0.749685
C	-0.036247	0.715571	0.111186
C	0.209116	-0.380020	1.146493
O	1.475256	-0.176564	1.768113
O	1.720925	3.480342	1.978415
O	-0.089609	3.053407	-0.275217
O	-1.347875	0.551286	-0.409295
C	0.245371	-1.780520	0.560000
O	1.314755	-1.947581	-0.360808
O	2.768423	1.055986	3.180581
O	2.401575	4.720383	-0.403990
H	2.260353	1.997697	0.655552
H	-0.660870	2.206688	1.512926
H	-0.381225	3.877429	0.167513
H	0.699973	0.614837	-0.697258
H	-1.552595	1.361251	-0.905808
H	-0.591310	-0.339200	1.901391
H	-0.678108	-1.975745	0.012175
H	0.328962	-2.505502	1.379237
H	2.132439	-1.690236	0.094747
H	3.465173	1.595148	2.740117
H	0.758328	1.135175	3.173412
H	2.211332	4.381865	0.504708
H	1.737503	4.251211	-0.935052
H	0.461101	4.472721	1.908912
O	-0.393211	4.981694	1.701513
H	-0.141354	5.901084	1.526281
H	3.416648	3.468471	2.132892
O	4.341821	3.117848	2.275839
H	4.792637	3.166728	1.420032
H	1.721176	3.502828	3.691790
O	1.789100	3.407633	4.682140
H	2.236752	2.549784	4.775043
H	-1.336877	3.852173	5.039800
O	-0.797223	3.436080	5.745538
H	0.118298	3.403650	5.387922
H	-1.616943	4.790903	2.934656
O	-2.262465	4.665671	3.672800
H	-2.969860	4.107636	3.317856

β-D-glucose$^{-}_{(aq)}$ (C3-O^{-}, 6w)

C	1.233133	1.859556	1.783161
C	1.441214	2.137862	0.296620
C	0.296401	1.548545	-0.542266
C	0.040550	0.085494	-0.160215
C	-0.074469	-0.091179	1.351402
O	1.072264	0.463674	1.992134
O	1.559979	3.540658	0.121370
O	0.535294	1.703289	-1.907452
O	-1.186882	-0.379402	-0.729129
C	-0.142137	-1.540715	1.801237
O	1.039916	-2.258623	1.472772
O	2.294174	2.286983	2.577171
O	-0.070786	4.224649	-2.080060
H	2.382516	1.652403	-0.006982
H	1.017963	3.837748	-0.645028
H	-0.616311	2.104600	-0.249114
H	0.866331	-0.533605	-0.531547
H	-1.065053	-0.455765	-1.711462
H	-0.981046	0.424703	1.705297
H	-0.968729	-2.044651	1.298497
H	-0.316760	-1.568921	2.884133
H	1.790644	-1.767405	1.843193
H	3.126523	1.962517	2.195763
H	0.346240	2.389982	2.152326
H	0.117684	3.240620	-2.201919
H	-0.989025	4.260113	-1.737310
H	2.043199	1.358487	-2.458918
O	2.954302	1.166965	-2.841045
H	2.846823	0.373739	-3.384801
H	-0.268160	0.715281	-2.933309
O	-0.772572	-0.035873	-3.378650
H	-1.640667	0.335142	-3.646903
H	-2.923456	3.104735	-0.937883
O	-2.707473	4.060617	-1.024765
H	-2.762584	4.427156	-0.130007
H	-3.506222	1.139572	-2.749978
O	-3.358523	1.042830	-3.718209
H	-4.028183	0.417637	-4.032844
H	-2.544624	0.710172	-0.746831
O	-3.294676	1.329066	-0.956990
H	-4.048186	1.055155	-0.411815

β-D-glucose$^{-}_{(aq)}$ (C4-O^{-}, 6w)

C	1.384080	2.016763	1.308903
C	0.125104	1.599313	0.563402
C	0.187160	0.116817	0.186568
C	0.500513	-0.683045	1.468150
O	1.720377	-0.236977	2.059719
C	1.643142	1.106052	2.499586
O	-0.014249	2.402792	-0.606685
O	-0.988507	-0.354745	-0.413688
C	0.650904	-2.173821	1.197338
O	-0.594457	-2.744160	0.820726
O	2.869006	1.470452	3.050326
O	1.197536	3.367059	1.709287
O	-1.907287	1.262319	-2.379779
H	1.406506	-2.332012	0.414139
H	2.246591	1.937883	0.633570
H	2.034101	3.705744	2.063849
H	-0.745431	1.763756	1.215420
H	0.106855	3.326673	-0.331568
H	1.042038	-0.015601	-0.499782

β-D-glucose$^{-}_{(aq)}$ (C6-O^{-}, 6w)

C	1.139636	1.660030	2.195426
C	0.653033	2.211976	0.860524
C	-0.690646	1.594592	0.517588
C	-0.614399	0.078024	0.578854
C	-0.099030	-0.364825	1.949499
O	1.182885	0.246982	2.149048
O	0.473159	3.616961	0.908147
O	-1.121105	1.935618	-0.793345
O	-1.903792	-0.482443	0.339372
C	0.061342	-1.877157	2.105347
O	0.787138	-2.476445	1.079429
O	2.432881	2.135831	2.397988
O	4.201856	-2.610775	-1.521692
O	1.695201	-4.901743	1.119449
O	-1.006855	-3.028007	-0.708125
H	1.388768	1.943355	0.090986
H	1.338460	4.035897	1.036773
H	-1.436422	1.944089	1.246519
H	-1.274239	2.892517	-0.830939

β-D-glucose$^{0}_{(g)}$

C	-0.322264	0.313562	0.035112
O	-0.131298	-0.379152	1.272136
C	1.143476	-0.192758	1.851549
C	2.225810	-0.716987	0.919218
C	2.117859	-0.008395	-0.417478
C	0.715772	-0.148736	-0.987114
O	3.524237	-0.464062	1.424017
O	3.017640	-0.540315	-1.373541
O	0.558706	0.657696	-2.139244
C	-1.751801	0.019543	-0.389553
O	-1.995001	-1.368820	-0.497885
H	2.063393	-1.794826	0.784240
H	3.624655	-0.932469	2.266582
H	2.330673	1.061139	-0.266153
H	3.913387	-0.494997	-1.004839
H	0.530582	-1.203375	-1.228813
H	1.260927	0.418889	-2.763984
H	-0.209298	1.397842	0.190531
H	-1.932529	0.452926	-1.374691

H	-1.019376	-2.062983	0.252685
H	-0.323866	-0.545342	2.186082
H	0.990974	-2.678394	2.105767
H	2.959996	1.078331	3.932411
H	0.836776	1.198858	3.242735
H	-1.523365	0.652945	-1.688362
H	-1.532993	2.115447	-2.105844
H	-3.984044	-1.306153	-2.600500
O	-3.651998	-2.140410	-3.001885
H	-2.679380	-2.118959	-2.868038
H	-0.826488	-1.369708	-1.749609
O	-0.863296	-1.973566	-2.545675
H	-0.488520	-2.818605	-2.256255
H	-2.369088	-0.120297	0.348950
O	-3.302456	0.045230	0.719761
H	-3.517640	-0.709621	1.287983
H	-3.368593	1.598881	1.588669
O	-3.395011	2.449631	2.084911
H	-3.473117	3.140289	1.411394
H	-4.228258	0.165631	-0.880966
O	-4.417093	0.307474	-1.834312
H	-3.624849	0.811419	-2.136472
H	0.086528	-0.278519	-0.185860
H	-2.322115	0.056474	-0.353960
H	-0.789470	-0.009929	2.730681
H	-0.957261	-2.296109	2.171251
H	0.532725	-2.039896	3.089529
H	2.686845	2.007226	3.325025
H	0.471285	1.978713	3.009493
H	3.627173	-3.409691	-1.554617
H	3.888623	-2.111706	-0.734380
H	1.291269	-3.971792	1.203935
H	0.977397	-5.528944	1.288855
H	-0.257070	-2.846818	-0.041934
H	-1.570729	-2.238903	-0.612327
H	1.705287	-4.543548	-2.039869
O	2.498609	-4.831735	-1.532802
H	2.203216	-4.914674	-0.593928
H	3.054441	-0.537004	1.075751
O	3.185064	-1.456919	0.797316
H	2.258885	-1.853879	0.903208
H	-0.286967	-3.512209	-2.157054
O	0.230629	-3.929624	-2.899102
H	0.461533	-3.211034	-3.505731
H	-2.438407	0.481112	0.333364
H	-1.760367	-1.774546	0.350968
O	1.215812	-0.930881	3.028597
H	0.572995	-0.587037	3.666700
H	1.296812	0.881374	2.049263

β-D-glucose$^{-}_{(g)}$ (C1-O^{-})

C	-0.368602	0.306084	0.044049
C	-0.103846	-0.470308	1.341863
O	1.194608	-0.223583	1.827059
C	1.459885	1.241234	2.162003
C	1.300691	1.946917	0.798245
C	-0.092592	1.781741	0.255318
C	-0.192472	-1.980984	1.146622
O	0.946482	-2.463434	0.452040
O	2.615104	1.469602	2.698074
O	1.600573	3.326434	0.967741
O	-0.293982	2.456764	-0.995371
O	-1.725567	0.097225	-0.351882
H	2.030252	1.495058	0.110885
H	2.286360	3.287114	1.670895
H	-0.815629	2.179878	0.982556
H	0.140321	3.319539	-0.907150
H	0.302155	-0.072772	-0.738353
H	-1.908652	0.749385	-1.045500
H	-0.869147	-0.162602	2.076785
H	-1.077481	-2.245480	0.561704
H	-0.259663	-2.459085	2.135023
H	1.685864	-1.935093	0.807837
H	0.573638	1.492192	2.807203

Sample input for calculations of C 1s vertical ionization energies using Q-Chem 4.3

```
$molecule
-1 1
 C   -0.341240    0.306568    0.050671
 C   -0.097323   -0.458165    1.350234
 O    1.199211   -0.210898    1.846571
 C    1.452838    1.228192    2.158488
 C    1.316800    1.954626    0.813743
 C   -0.058964    1.781336    0.235872
 C   -0.201080   -1.959226    1.166967
 O    0.866173   -2.451645    0.372849
 O    2.610235    1.435415    2.718883
 O    1.598810    3.327993    1.024716
 O   -0.222948    2.399836   -1.038725
 O   -1.687394    0.095033   -0.347952
 H    2.057677    1.519684    0.131842
 H    2.268487    3.289641    1.728360
 H   -0.795219    2.195011    0.934089
 H   -0.047902    3.339744   -0.934004
 H    0.339279   -0.081819   -0.713611
 H   -1.866733    0.709703   -1.067119
 H   -0.860947   -0.142020    2.074477
 H   -1.131563   -2.215958    0.665073
 H   -0.193924   -2.433452    2.152735
 H    1.655707   -2.000552    0.696279
 H    0.568320    1.506781    2.787205
$end

$rem
METHOD CAM-B3LYP
BASIS General
MAX_SCF_CYCLES 129
solvent_method  pcm
$end

$basis
H 0
cc-pVTZ
****
C 0
aug-cc-pCVTZ
****
O 0
aug-cc-pCVTZ
****
$end

$pcm
ChargeSeparation  Marcus
RADII            UFF
vdwScale         1.1
THEORY           IEFPCM
$end

@@@
$molecule
0 2
READ
$end
$rem
METHOD CAM-B3LYP
BASIS General
MAX_SCF_CYCLES 129
unrestricted TRUE
mom_start   1
scf_guess read
MAX_SCF_CYCLES 129
solvent_method  pcm
```

```
PCM_PRINT      1
$end

$basis
H 0
cc-pVTZ
****
C 0
aug-cc-pCVTZ
****
O 0
aug-cc-pCVTZ
****
$end

$pcm
stateSpecific  ground
RADII             UFF
vdwScale          1.1
THEORY            IEFPCM
$end

$occupied
 1 2 3 4 5 6 7 8 9 10 11 12 13 14 15 16 17 18 19 20 21 22 23 24 25 26 27 28 29 30 31 32 33 34 35 36 37 38 39 40 41 42 43 44 45 46 47 48
 1 2 3 4 5 6 8 9 10 11 12 13 14 15 16 17 18 19 20 21 22 23 24 25 26 27 28 29 30 31 32 33 34 35 36 37 38 39 40 41 42 43 44 45 46 47 48
$end
```

Cartesian coordinates of the structures used to calculate C 1s vertical ionization energies. Optimization was performed on the CAM-B3LYP/aug-cc-pVTZ level of theory using the polarizable continuum model (PCM). Minima were confirmed by the absence of imaginary vibrational frequencies.

α-D-glucose$^0_{(aq)}$

O	-0.286971	0.361210	0.029241
C	-0.130762	-0.265603	1.275660
C	1.293723	-0.111994	1.792918
C	1.698199	1.351453	1.789446
C	1.489795	1.953586	0.418759
C	0.048332	1.749994	-0.020702
H	-0.366670	-1.314496	1.100964
O	-0.974250	0.276061	2.258794
H	-1.873882	-0.034133	2.119154
H	1.962024	-0.662309	1.131278
O	1.439168	-0.679204	3.079258
H	0.677569	-0.396094	3.600202
H	1.077281	1.897005	2.507491
O	3.067256	1.510475	2.118709
H	3.208675	1.130764	2.991968
H	2.151309	1.449421	-0.292417
O	1.748368	3.345160	0.428376
H	2.606128	3.474470	0.846479
H	-0.607645	2.317560	0.644179
C	-0.209938	2.183548	-1.446501
H	0.068967	3.228386	-1.559088
H	-1.278438	2.084617	-1.654484
O	0.555228	1.442477	-2.378848
H	0.372278	0.510306	-2.221498

α-D-glucose$^-_{(aq)}$ (C1-O$^-$)

C	-0.278989	0.347314	0.027059
O	-0.132334	-0.319867	1.261536
C	1.205095	-0.191039	1.901996
C	2.234398	-0.773910	0.909486
C	2.088780	-0.125549	-0.451675
C	0.685945	-0.248881	-0.987609
O	1.545320	1.025175	2.260932
O	3.523664	-0.475629	1.428826
O	2.973288	-0.698609	-1.412627
O	0.553091	0.445836	-2.220127
C	-1.731986	0.182741	-0.368575
O	-2.120146	-1.181920	-0.340209
H	1.073436	-0.910228	2.737301
H	2.140125	-1.857683	0.814697
H	3.322544	0.354169	1.910710
H	2.315115	0.939024	-0.340971
H	3.863774	-0.620765	-1.055388
H	0.441659	-1.307305	-1.127681
H	1.326073	0.221944	-2.749102
H	-0.058779	1.415176	0.131149
H	-1.890641	0.550403	-1.380402
H	-2.351423	0.769468	0.316308
H	-1.723754	-1.542133	0.462750

α-D-glucose$^-_{(aq)}$ (C2-O$^-$)

C	-0.280520	0.368966	-0.014083
O	-0.125089	-0.216660	1.275276
C	1.162852	-0.226240	1.849042
C	2.274524	-0.710860	0.901940
C	2.077993	-0.066774	-0.468916
C	0.686758	-0.266367	-1.003542
O	1.536827	1.052259	2.295233
O	3.504124	-0.378645	1.404029
O	3.032133	-0.597740	-1.381013
O	0.506806	0.367461	-2.265572
C	-1.738178	0.177635	-0.372689
O	-2.118966	-1.188966	-0.354241
H	1.061381	-0.902910	2.701565
H	2.513611	1.002778	2.157613
H	2.130159	-1.799638	0.766555
H	2.250569	1.010874	-0.366632
H	3.843093	-0.664030	-0.856404
H	0.468404	-1.334434	-1.092905
H	1.272859	0.151720	-2.807201
H	-0.074186	1.442777	0.029092
H	-1.920329	0.549255	-1.377879
H	-2.349914	0.752742	0.327152
H	-1.822100	-1.553369	0.486717

α-D-glucose$^-_{(aq)}$ (C3-O$^-$)

C	-0.262598	0.357223	0.032777
O	-0.076097	-0.262387	1.314857
C	1.223942	-0.191545	1.874465
C	2.221668	-0.771708	0.885265
C	2.139083	-0.062465	-0.463076
C	0.713525	-0.237639	-0.967676
O	1.574779	1.125628	2.201801
O	3.554436	-0.714340	1.352598
O	3.087009	-0.507791	-1.338807
O	0.590108	0.404935	-2.228193
C	-1.722055	0.151369	-0.306014
O	-2.086085	-1.220427	-0.329568
H	1.161137	-0.797951	2.779250
H	1.043166	1.406618	2.952553
H	1.933813	-1.820399	0.741129
H	4.055643	-0.699546	0.517240
H	2.253678	1.021835	-0.257305
H	0.503449	-1.306759	-1.082018
H	1.474364	0.292213	-2.613251
H	-0.077670	1.432266	0.110552
H	-1.923005	0.553507	-1.296494
H	-2.335785	0.693475	0.418519
H	-1.768637	-1.612565	0.490831

α-D-glucose$^-_{(aq)}$ (C4-O$^-$)

C	-0.281720	0.317192	0.033154
O	-0.081322	-0.258577	1.330684
C	1.224861	-0.170864	1.826404
C	2.275008	-0.734705	0.861184
C	2.084337	-0.139543	-0.516494
C	0.647235	-0.343399	-0.984490
O	1.604274	1.162935	2.094407
O	3.574009	-0.534591	1.395381
O	2.952863	-0.740603	-1.464154
O	0.474686	0.111877	-2.272218
C	-1.735323	0.112043	-0.347393
O	-1.958333	0.616035	-1.649217
H	1.222177	-0.748278	2.750719
H	1.147654	1.470707	2.882732
H	2.135573	-1.813554	0.799527
H	3.618862	0.381502	1.693010
H	2.286116	0.937827	-0.475074
H	2.480280	-0.595993	-2.301034
H	0.449352	-1.433500	-0.904603
H	-0.071050	1.389866	0.072839
H	-2.393168	0.625412	0.357191
H	-1.963534	-0.960108	-0.292558
H	-1.076425	0.463437	-2.121291

α-D-glucose$^-_{(aq)}$ (C6-O$^-$)

C	-0.304901	0.322122	0.028646
O	-0.112131	-0.301846	1.311003
C	1.158337	-0.195927	1.873405
C	2.228083	-0.748968	0.939642
C	2.105794	-0.112475	-0.431699
C	0.695566	-0.251523	-0.960635
O	1.528805	1.137213	2.160374
O	3.527655	-0.572482	1.475427
O	2.982609	-0.728077	-1.367153
O	0.565630	0.443163	-2.194293
C	-1.767852	0.109907	-0.385882
O	-2.131474	-1.169793	-0.659260
H	1.121659	-0.776840	2.795239
H	1.045329	1.443803	2.932990
H	2.075752	-1.823461	0.844006
H	3.569799	0.324073	1.830016
H	2.346267	0.952625	-0.355342
H	3.884724	-0.627201	-1.047203
H	0.464896	-1.310378	-1.104279
H	1.299440	0.169314	-2.754153
H	-0.124126	1.397254	0.130469
H	-1.909097	0.805516	-1.241284
H	-2.349751	0.571509	0.443969

β-D-glucose$^0_{(aq)}$

C	-0.322729	0.297615	0.040413
O	-0.140681	-0.411811	1.260710
C	1.127425	-0.212341	1.853635
C	2.217479	-0.716712	0.925140
C	2.105761	-0.022729	-0.413680
C	0.709025	-0.165237	-0.979086
O	3.504632	-0.442391	1.438332
O	3.001873	-0.561825	-1.368443
O	0.555752	0.621607	-2.144432
C	-1.748294	0.035264	-0.393731
O	-2.008650	-1.346111	-0.559193
H	2.071209	-1.793401	0.792509

β-D-glucose$^-_{(aq)}$ (C1-O$^-$)

C	-0.341240	0.306568	0.050671
C	-0.097323	-0.458165	1.350234
O	1.199211	-0.210898	1.846571
C	1.452838	1.228192	2.158488
C	1.316800	1.954626	0.813743
C	-0.058964	1.781336	0.235872
C	-0.201080	-1.959226	1.166967
O	0.866173	-2.451645	0.372849
O	2.610235	1.435415	2.718883
O	1.598810	3.327993	1.024716
O	-0.222948	2.399836	-1.038725
O	-1.687394	0.095033	-0.347952

β-D-glucose$^-_{(aq)}$ (C2-O$^-$)

C	-0.100345	1.782824	0.266080
C	-0.367995	0.314675	0.047978
C	-0.119776	-0.452606	1.350763
O	1.164770	-0.171926	1.898958
C	1.370722	1.212605	2.124220
C	1.307009	1.976259	0.810342
O	-1.716127	0.058332	-0.331350
C	-0.167105	-1.954065	1.168470
O	0.883448	-2.416064	0.337126
O	2.632785	1.387903	2.686630
O	1.673702	3.272771	0.925385
O	-0.226746	2.520654	-0.938470

H	3.606057	-0.918310	2.268556
H	2.315240	1.043427	-0.269773
H	3.898908	-0.439176	-1.042287
H	0.527022	-1.220006	-1.204536
H	1.288643	0.410703	-2.732452
H	-0.198769	1.374357	0.205617
H	-1.925759	0.514059	-1.353693
H	-2.424990	0.473328	0.344329
H	-1.761901	-1.784088	0.262149
O	1.193924	-0.951508	3.024098
H	0.713679	-0.495527	3.721920
H	1.266915	0.857538	2.052880

H	2.057677	1.519684	0.131842
H	2.268487	3.289641	1.728360
H	-0.795219	2.195011	0.934089
H	-0.047902	3.339744	-0.934004
H	0.339279	-0.081819	-0.713611
H	-1.866733	0.709703	-1.067119
H	-0.860947	-0.142020	2.074477
H	-1.131563	-2.215958	0.665073
H	-0.193924	-2.433452	2.152735
H	1.655707	-2.000552	0.696279
H	0.568320	1.506781	2.787205

H	1.977528	1.393617	0.132584
H	-0.823955	2.164226	1.000203
H	0.382666	3.262961	-0.776978
H	0.311098	-0.062627	-0.721150
H	-1.916974	0.631204	-1.077621
H	-0.901570	-0.162252	2.063647
H	-1.104422	-2.235173	0.694406
H	-0.117199	-2.429779	2.151819
H	1.694573	-2.019104	0.673204
H	2.901724	2.241079	2.296616
H	0.593512	1.563712	2.821400

β-D-glucose$^{-}_{(aq)}$ (C3-O^{-})

C	-0.261651	0.303502	0.001354
C	-0.162237	-0.431232	1.325785
O	1.077220	-0.102358	1.962854
C	1.329555	1.279813	2.201887
C	1.261273	2.023486	0.886027
C	-0.102380	1.796499	0.240502
C	-0.168721	-1.939495	1.211002
O	0.957513	-2.424518	0.499929
O	2.557157	1.371107	2.848689
O	1.432685	3.416634	1.039025
O	-0.287913	2.551435	-0.867700
O	-1.520008	0.088185	-0.614253
H	2.046811	1.615427	0.231795
H	0.895418	3.745767	0.291625
H	-0.845732	2.040991	1.041263
H	0.539648	-0.046931	-0.659474
H	-1.634329	0.914131	-1.116906
H	-1.002529	-0.127266	1.960966
H	-1.057323	-2.254145	0.666883
H	-0.204904	-2.375362	2.213712
H	1.728619	-1.987205	0.877252
H	3.254157	1.202532	2.205066
H	0.586680	1.672281	2.902461

β-D-glucose$^{-}_{(aq)}$ (C4-O^{-})

C	-0.313888	0.283656	0.004312
C	-0.108982	-0.430441	1.344649
O	1.209301	-0.183754	1.840864
C	1.452127	1.176320	2.064235
C	1.321212	1.983224	0.778421
C	-0.061003	1.770832	0.222207
C	-0.294470	-1.926307	1.186868
H	0.511481	-2.308087	0.544987
O	2.747339	1.258242	2.585591
O	1.603651	3.332333	1.129361
O	-0.239110	2.448544	-1.008686
O	-1.543033	0.076881	-0.562973
H	2.068372	1.615766	0.068464
H	1.539989	3.869740	0.334533
H	-0.793611	2.143098	0.951221
H	-0.949552	1.927362	-1.421444
H	0.517829	-0.074485	-0.643353
H	-1.741913	-1.380286	0.047467
H	-0.848356	-0.055151	2.064098
O	-1.566287	-2.185586	0.635556
H	-0.204657	-2.427685	2.153925
H	3.050222	2.162036	2.439289
H	0.725664	1.560346	2.796912

β-D-glucose$^{-}_{(aq)}$ (C6-O^{-})

C	-0.304529	0.263528	0.053580
C	-0.095300	-0.516341	1.343845
O	1.188638	-0.178889	1.883418
C	1.329775	1.180145	2.204247
C	1.227331	2.021540	0.944313
C	-0.098189	1.747851	0.272729
C	-0.161905	-2.039281	1.154799
O	0.666894	-2.558785	0.212646
O	2.595508	1.389436	2.748900
O	1.286236	3.408119	1.226524
O	-0.193220	2.386888	-0.993162
O	-1.623977	0.023375	-0.416512
H	2.045670	1.729816	0.279137
H	2.139636	3.601663	1.626881
H	-0.896891	2.117661	0.925464
H	-0.113604	3.336843	-0.862086
H	0.422196	-0.097465	-0.677722
H	-1.774554	0.612773	-1.162692
H	-0.869968	-0.220579	2.065202
H	-1.237960	-2.241736	0.963265
H	0.016928	-2.441898	2.177645
H	2.613374	1.065192	3.654384
H	0.549597	1.475900	2.918392

n-D-glucose$^{0}_{(aq)}$

C	0.025572	0.264147	0.003879
C	-0.057019	-0.002800	1.494254
H	1.058847	0.417350	-0.291511
H	-0.362108	-0.602621	-0.544148
O	-0.687791	1.437083	-0.345309
H	-1.570484	1.374366	0.034549
H	0.314576	0.873492	2.026434
O	-1.418927	-0.133456	1.891484
C	0.744888	-1.223476	1.917062
H	-1.830573	-0.860247	1.408511
C	0.803054	-1.441034	3.429793
H	0.307405	-2.119923	1.459118
O	2.063025	-1.035168	1.434921
H	2.623278	-1.679702	1.883463
C	-0.481143	-1.925245	4.096810
O	1.802705	-2.433861	3.608538
H	1.125048	-0.510237	3.910818
H	1.729609	-2.759904	4.513636
C	-1.541272	-0.869501	4.358066
H	-0.921147	-2.753453	3.539539
O	-0.075104	-2.358905	5.398322
H	-0.759374	-2.919263	5.777612
O	-2.703743	-1.154999	4.486008
H	-1.176082	0.148667	4.561930

n-D-glucose$^{-}_{(aq)}$ (C2-O^{-})

C	-0.251221	0.201343	-0.187876
O	-0.572092	-0.054015	1.170129
C	1.223761	0.508697	-0.353715
C	1.596121	0.822794	-1.794727
O	0.828694	1.945948	-2.195501
O	1.998543	-0.563622	0.179896
C	3.094154	1.136977	-1.972436
O	3.249163	2.048519	-3.037508
C	3.940226	-0.122202	-2.387353
O	4.184671	-0.066851	-3.738230
C	5.285159	-0.105752	-1.713643
O	5.742808	-0.986880	-1.020857
H	-0.849899	1.049513	-0.508351
H	-0.514764	-0.660608	-0.811982
H	0.090770	-0.666258	1.508477
H	1.475293	1.373465	0.262037
H	1.877203	-1.347311	-0.367191
H	1.337557	-0.035812	-2.429828
H	1.400920	2.401283	-2.835217
H	3.472879	1.590796	-1.051930
H	3.662152	1.406581	-3.716948
H	3.418293	-1.034634	-2.066700
H	5.904146	0.774986	-1.971987

n-D-glucose$^{-}_{(aq)}$ (C3-O^{-})

C	-0.237750	0.281273	-0.169780
C	1.215000	0.700615	-0.271919
O	1.955687	0.076067	0.779667
O	-0.757329	0.550580	1.124190
C	1.824191	0.388445	-1.635790
C	3.223220	1.024276	-1.811378
C	4.081552	0.165767	-2.839150
C	4.937868	-0.792688	-2.090717
O	4.614411	-1.919045	-1.790921
O	1.019964	0.914575	-2.670859
O	3.105181	2.281909	-2.331081
O	4.869555	1.079931	-3.553255
H	-0.841738	0.832325	-0.884560
H	-0.324136	-0.787688	-0.399112
H	-0.091983	0.257119	1.757085
H	1.291812	1.771410	-0.077420
H	2.038281	-0.864958	0.588277
H	1.876077	-0.701103	-1.743005
H	1.534653	1.732146	-2.890822
H	3.735734	1.010343	-0.837832
H	3.407600	-0.386033	-3.501613
H	4.370917	1.921596	-3.331048
H	5.914379	-0.381718	-1.773918

n-D-glucose$^{-}_{(aq)}$ (C4-O^{-})

C	-0.044768	0.193335	-0.078030
C	1.348596	0.694299	-0.386315
O	2.291371	-0.016267	0.432194
O	-0.328974	0.271562	1.313872

n-D-glucose$^{-}_{(aq)}$ (C6-O^{-})

C	0.106491	0.213776	0.082804
O	0.246679	-0.705090	1.101374
C	1.534053	0.565372	-0.434632
C	1.599369	0.874222	-1.911464

α-D-glucose$^{0}_{(aq)}$ (1w)

C	0.180033	1.776660	-0.089899
O	0.010714	0.361016	-0.151383
C	0.164282	-0.331399	1.069804
C	1.536483	-0.055367	1.672596

C	1.692699	0.580267	-1.872209
C	3.125492	1.212956	-2.149602
C	4.137643	0.190579	-2.666407
C	4.419608	-0.868666	-1.628194
O	3.953160	-1.980715	-1.630561
O	0.782650	1.210249	-2.674741
O	2.918715	2.214146	-3.106253
O	5.359334	0.864613	-2.943272
H	-0.781340	0.797283	-0.598611
H	-0.142925	-0.842154	-0.426783
H	0.456338	-0.046141	1.773828
H	1.431724	1.736849	-0.072689
H	2.327228	-0.931000	0.129706
H	1.754869	-0.505405	-2.083806
H	3.535545	1.648653	-1.232297
H	1.905868	2.077548	-3.221353
H	3.736492	-0.282203	-3.565355
H	5.917707	0.302460	-3.488199
H	5.125863	-0.552257	-0.840946

O	0.765439	2.001115	-2.149774
O	2.305749	-0.579895	-0.151004
C	2.998109	1.185924	-2.427902
O	2.819373	1.581222	-3.783222
C	3.988624	0.029566	-2.370442
O	5.074304	0.422214	-3.216762
C	4.610932	-0.232193	-1.007814
O	5.252720	-1.231740	-0.799773
H	-0.399547	1.141263	0.389537
H	-0.471952	-0.186334	-0.772801
H	1.922900	1.430551	0.121055
H	1.661130	-0.983559	0.546778
H	1.219818	0.007768	-2.465820
H	0.930728	2.291775	-3.053240
H	3.405740	2.029595	-1.858115
H	3.685249	1.561501	-4.207273
H	3.535232	-0.891809	-2.738131
H	5.648670	-0.335766	-3.362992
H	4.577722	0.586769	-0.275963

C	1.766622	1.440607	1.784641
C	1.563733	2.110890	0.445969
O	-0.787003	0.033097	2.019211
O	1.671696	-0.682706	2.932969
O	3.087189	1.732249	2.211147
O	1.661369	3.518881	0.561037
C	-0.054737	2.280639	-1.496606
O	0.831055	1.690243	-2.430459
H	0.065897	-1.384582	0.805174
H	-1.620813	-0.449725	1.843770
H	2.300411	-0.482650	1.023481
H	0.837375	-0.539499	3.398120
H	1.046467	1.858725	2.495473
H	3.223343	1.304011	3.062565
H	2.314826	1.734885	-0.255665
H	2.468231	3.709740	1.050778
H	-0.570422	2.216955	0.571852
H	0.113060	3.354503	-1.527586
H	-1.094601	2.083287	-1.770164
H	0.757874	0.735787	-2.324964
O	-3.062627	-1.360462	1.517154
H	-2.963180	-2.313574	1.606549
H	-3.417698	-1.221981	0.633355

α-D-glucose$^{-}_{(aq)}$ (C1-O^{-}, 1w)

C	0.694032	-0.203177	-1.018262
C	-0.266619	0.327861	0.035313
O	-0.118700	-0.419821	1.226452
C	1.204606	-0.341145	1.853912
C	2.234872	-0.864186	0.838306
C	2.099784	-0.126922	-0.478997
C	-1.720900	0.196316	-0.364047
O	-2.112676	-1.163753	-0.458864
O	1.551152	0.872258	2.275378
O	3.530372	-0.637960	1.374468
O	2.974336	-0.652044	-1.473383
O	0.561022	0.576328	-2.197921
H	1.091024	-1.079952	2.666887
H	2.118730	-1.936456	0.671031
H	3.383849	0.173957	1.894866
H	2.339403	0.926095	-0.303173
H	3.868329	-0.601046	-1.120092
H	0.443627	-1.247992	-1.229689
H	1.326126	0.380815	-2.749094
H	-0.041753	1.384375	0.211568
H	-1.883247	0.652777	-1.338199
H	-2.335756	0.720992	0.373026
H	-1.750162	-1.595698	0.323542
H	1.129508	1.014698	3.773947
O	0.867616	1.062069	4.750720
H	-0.091238	1.027559	4.748958

β-D-glucose$^{0}_{(aq)}$ (1w)

C	0.741251	-0.300986	-1.193042
C	-0.316233	0.228638	-0.233564
O	-0.155241	-0.376052	1.042728
C	1.103273	-0.112727	1.647097
C	2.210601	-0.681011	0.777211
C	2.124228	-0.096611	-0.613286
C	-1.729475	-0.084236	-0.675159
O	-1.974550	-1.477849	-0.721499
O	1.153338	-0.731594	2.875800
O	3.485522	-0.363459	1.297837
O	3.046247	-0.697446	-1.506015
O	0.603725	0.388410	-2.421039
H	2.070521	-1.765900	0.727488
H	3.529733	-0.710428	2.194914
H	2.318734	0.980148	-0.550165
H	3.934834	-0.538640	-1.172711
H	0.575409	-1.372504	-1.337774
H	1.354264	0.144289	-2.972824
H	-0.206132	1.316991	-0.155296
H	-1.890092	0.305202	-1.677569
H	-2.426356	0.410166	0.006550
H	-1.728756	-1.838049	0.137252
H	0.727170	-0.158853	3.545854
H	1.223246	0.974796	1.744742
O	0.000507	0.908009	4.711597
H	-0.905672	1.155199	4.501922
H	0.469641	1.738732	4.838135

β-D-glucose$^{-}_{(aq)}$ (C1-O^{-}, 1w)

C	-0.014909	1.782468	0.221665
C	-0.220659	0.294339	0.042875
C	-0.096655	-0.429248	1.381398
O	1.128932	-0.116519	2.010706
C	1.294285	1.317622	2.300498
C	1.281819	2.025788	0.942414
O	-1.505074	0.021149	-0.494307
C	-0.123949	-1.936841	1.233984
O	1.026722	-2.410101	0.553827
O	2.396100	1.565328	2.984365
O	1.465065	3.416284	1.152410
O	-0.056967	2.367878	-1.076169
H	2.113288	1.622187	0.353871
H	2.080679	3.447972	1.900386
H	-0.838388	2.180923	0.825335
H	0.064585	3.316782	-0.976436
H	0.554845	-0.083730	-0.630390
H	-1.624657	0.606390	-1.249576
H	-0.944077	-0.125284	2.010671
H	-0.994294	-2.241325	0.656964
H	-0.191535	-2.384348	2.229678
H	1.775449	-1.948783	0.949743
H	0.366739	1.600619	2.846994
H	3.692270	1.068520	2.205397
O	4.523229	0.761600	1.721290
H	4.314769	-0.129574	1.432662

α-D-glucose$^{0}_{(g)}$

O	-0.295947	0.364402	0.042105
C	-0.119481	-0.268426	1.275786
C	1.301294	-0.102920	1.796970
C	1.692735	1.362517	1.796529
C	1.484872	1.965642	0.426390
C	0.045887	1.755655	-0.016316
H	-0.341235	-1.320873	1.094203
O	-0.969744	0.247842	2.273646
H	-1.878505	0.013906	2.068188
H	1.976734	-0.645002	1.135144
O	1.454397	-0.670613	3.081239
H	0.682672	-0.410064	3.597229
H	1.060091	1.902541	2.509968
O	3.055798	1.527416	2.136028
H	3.215114	1.045309	2.953512

α-D-glucose$^{-}_{(g)}$ (C1-O^{-})

C	-0.287649	0.336285	0.021165
O	-0.139340	-0.347227	1.239799
C	1.217946	-0.190669	1.896660
C	2.229389	-0.792756	0.891589
C	2.069619	-0.116791	-0.453005
C	0.676311	-0.250770	-1.003346
O	1.564059	1.018379	2.210917
O	3.531160	-0.496538	1.381636
O	2.998175	-0.632734	-1.413447
O	0.556740	0.448040	-2.239867
C	-1.743129	0.171126	-0.376866
O	-2.148649	-1.180844	-0.247926
H	1.069587	-0.899837	2.741431
H	2.130368	-1.876276	0.788739
H	3.323857	0.330809	1.872927

β-D-glucose$^{0}_{(g)}$

C	-0.321073	0.309714	0.039530
O	-0.131823	-0.380619	1.272271
C	1.140927	-0.197811	1.846454
C	2.218315	-0.717327	0.914580
C	2.108787	-0.012181	-0.416841
C	0.711308	-0.147725	-0.982032
O	3.513359	-0.467642	1.419427
O	3.006981	-0.540547	-1.371206
O	0.555474	0.657122	-2.130909
C	-1.742977	0.016212	-0.390833
O	-1.981926	-1.368631	-0.505768
H	2.049847	-1.790304	0.777957
H	3.599074	-0.920435	2.263256
H	2.316640	1.053841	-0.260920
H	3.891453	-0.508844	-0.994348

H	2.150742	1.469741	-0.286836
O	1.722955	3.357076	0.442292
H	2.590710	3.496019	0.833622
H	-0.610343	2.328452	0.644052
C	-0.199994	2.176794	-1.448920
H	0.110437	3.211899	-1.569559
H	-1.271845	2.105823	-1.658382
O	0.546507	1.406396	-2.364974
H	0.331894	0.483129	-2.202578
H	2.266671	0.947954	-0.298459
H	3.841511	-0.648404	-0.948100
H	0.432261	-1.308333	-1.152632
H	1.381387	0.287511	-2.708565
H	-0.058725	1.400620	0.142484
H	-1.886237	0.471659	-1.414453
H	-2.356591	0.813019	0.265486
H	-1.664467	-1.486490	0.532218
H	0.524534	-1.199234	-1.218615
H	1.264678	0.430603	-2.740149
H	-0.208293	1.390646	0.189458
H	-1.915814	0.457043	-1.369773
H	-2.429537	0.475647	0.327015
H	-1.728315	-1.772646	0.329799
O	1.215934	-0.933599	3.020725
H	0.614162	-0.559664	3.670023
H	1.300057	0.873104	2.039880

β-D-glucose$^{-}_{(g)}$ (C1-O^{-})

C	-0.367715	0.305242	0.054998
C	-0.098463	-0.468524	1.348242
O	1.191812	-0.224144	1.836191
C	1.454670	1.253676	2.161849
C	1.298060	1.940776	0.793886
C	-0.089932	1.776172	0.255564
C	-0.185061	-1.972319	1.138707
O	0.951758	-2.439257	0.438324
O	2.593722	1.490200	2.696851
O	1.614038	3.312553	0.956085
O	-0.293826	2.445592	-0.994197
O	-1.722192	0.096526	-0.336956
H	2.019503	1.468318	0.117020
H	2.274151	3.253930	1.674248
H	-0.805627	2.172794	0.985284
H	0.161997	3.288725	-0.911128
H	0.303407	-0.074701	-0.721483
H	-1.903725	0.754227	-1.014660
H	-0.868609	-0.167880	2.075535
H	-1.071772	-2.226382	0.558587
H	-0.253341	-2.456583	2.119805
H	1.671875	-1.891378	0.785088
H	0.553570	1.498263	2.789804

Sample input for calculations of pK_a values according to Thapa and Schlegel using Gaussian 09 (revision D.01)

```
%mem=24GB
%nProc=16
#p wB97XD/6-31+g* nosymm opt freq scrf=(smd,solvent=water)

text

-1,1
 C   -0.341240    0.306568    0.050671
 C   -0.097323   -0.458165    1.350234
 O    1.199211   -0.210898    1.846571
 C    1.452838    1.228192    2.158488
 C    1.316800    1.954626    0.813743
 C   -0.058964    1.781336    0.235872
 C   -0.201080   -1.959226    1.166967
 O    0.866173   -2.451645    0.372849
 O    2.610235    1.435415    2.718883
 O    1.598810    3.327993    1.024716
 O   -0.222948    2.399836   -1.038725
 O   -1.687394    0.095033   -0.347952
 H    2.057677    1.519684    0.131842
 H    2.268487    3.289641    1.728360
 H   -0.795219    2.195011    0.934089
 H   -0.047902    3.339744   -0.934004
 H    0.339279   -0.081819   -0.713611
 H   -1.866733    0.709703   -1.067119
 H   -0.860947   -0.142020    2.074477
 H   -1.131563   -2.215958    0.665073
 H   -0.193924   -2.433452    2.152735
 H    1.655707   -2.000552    0.696279
 H    0.568320    1.506781    2.787205
```

Cartesian coordinates of the structures used to calculate pK_a values according to Thapa and Schlegel [1]. Optimization was performed on the ωB97XD/6-31+G* level of theory using the solvation model based on solute electron density (SMD). Minima were confirmed by the absence of imaginary vibrational frequencies.

α-D-glucose$^{0}_{(aq)}$

O	-0.279277	0.335269	0.027052
C	-0.153525	-0.281759	1.290630
C	1.275192	-0.133284	1.820179
C	1.708587	1.332334	1.814642
C	1.500598	1.933028	0.430206
C	0.051500	1.727965	-0.016375
H	-0.390396	-1.333944	1.116765
O	-1.030659	0.270049	2.242196
H	-1.925205	-0.056153	2.067821
H	1.935344	-0.698640	1.156596
O	1.416502	-0.720070	3.102600
H	0.776300	-0.299173	3.697499
H	1.109404	1.898303	2.541236
O	3.088133	1.460345	2.136457
H	3.208129	1.205483	3.062531
H	2.173480	1.429889	-0.277084
O	1.753960	3.330877	0.428927
H	2.680455	3.466959	0.674743
H	-0.612689	2.289128	0.654408
C	-0.228243	2.191732	-1.435687
H	-0.041118	3.264329	-1.511764
H	-1.286739	2.009861	-1.658380
O	0.597678	1.564639	-2.408249
H	0.408331	0.615492	-2.397613

α-D-glucose$^{-}_{(aq)}$ (C1-O^{-})

C	-0.272850	0.331013	0.049264
O	-0.112517	-0.327169	1.297715
C	1.204650	-0.155331	1.924842
C	2.246012	-0.750529	0.947911
C	2.128077	-0.117251	-0.436321
C	0.709566	-0.254798	-0.967074
O	1.495409	1.113175	2.264194
O	3.550150	-0.539786	1.482296
O	3.003544	-0.739851	-1.377705
O	0.544916	0.444207	-2.196176
C	-1.730369	0.183572	-0.356470
O	-2.127980	-1.169718	-0.555664
H	1.112277	-0.834204	2.793277
H	2.110612	-1.833429	0.859010
H	3.506902	0.349159	1.880412
H	2.375485	0.950929	-0.368536
H	3.913807	-0.538178	-1.118202
H	0.485962	-1.320513	-1.115013
H	1.191467	0.092194	-2.824931
H	-0.071150	1.407103	0.156881
H	-1.905431	0.699080	-1.302858
H	-2.356866	0.651577	0.413113
H	-1.997419	-1.643520	0.278420

α-D-glucose$^{-}_{(aq)}$ (C2-O^{-})

C	-0.266868	0.355451	0.018821
O	-0.101540	-0.261584	1.298624
C	1.187127	-0.209765	1.866463
C	2.296861	-0.715467	0.924296
C	2.121404	-0.073848	-0.459549
C	0.710397	-0.255562	-0.986919
O	1.485986	1.114426	2.267197
O	3.550757	-0.435723	1.451715
O	3.039074	-0.652351	-1.386843
O	0.513594	0.410159	-2.231559
C	-1.729737	0.183549	-0.354527
O	-2.114540	-1.175099	-0.529499
H	1.116427	-0.851371	2.751514
H	2.458857	1.166411	2.168219
H	2.120046	-1.802206	0.796975
H	2.328003	1.004553	-0.381266
H	3.896481	-0.673617	-0.931777
H	0.496567	-1.326473	-1.103421
H	1.125609	0.024275	-2.874329
H	-0.069170	1.433557	0.097736
H	-1.924041	0.686018	-1.303831
H	-2.347128	0.656740	0.418907
H	-2.001356	-1.632240	0.316190

α-D-glucose$^{-}_{(aq)}$ (C3-O^{-})

C	-0.259158	0.340657	0.055584
O	-0.061725	-0.292456	1.330193
C	1.239383	-0.182850	1.879320
C	2.257930	-0.748181	0.892378
C	2.160190	-0.072061	-0.479845
C	0.716324	-0.248889	-0.960644
O	1.567951	1.151561	2.184313
O	3.571578	-0.618662	1.417508
O	3.092762	-0.576210	-1.375081
O	0.536815	0.406907	-2.212380
C	-1.726489	0.161805	-0.293773
O	-2.107533	-1.196632	-0.482328
H	1.201324	-0.777226	2.796543
H	1.057021	1.420378	2.961389
H	2.021510	-1.815770	0.770729
H	4.152035	-0.718198	0.642415
H	2.308933	1.017378	-0.323540
H	0.511742	-1.323212	-1.081546
H	1.366941	0.253429	-2.695445
H	-0.073223	1.419455	0.149836
H	-1.936467	0.675410	-1.234484
H	-2.338299	0.621220	0.492663
H	-1.981675	-1.664306	0.355631

α-D-glucose$^{-}_{(aq)}$ (C4-O^{-})

C	-0.288008	0.308745	0.037496
O	-0.093526	-0.273784	1.334264
C	1.215024	-0.145028	1.846977
C	2.256102	-0.742532	0.888074
C	2.096620	-0.155616	-0.506830
C	0.654654	-0.330179	-0.996687
O	1.562717	1.201567	2.076280
O	3.567101	-0.591569	1.413421
O	2.999668	-0.811554	-1.393056
O	0.472775	0.197977	-2.274633
C	-1.748931	0.081303	-0.331723
O	-2.024631	0.664549	-1.600222
H	1.207626	-0.697451	2.789824
H	1.088380	1.515162	2.859543
H	2.080249	-1.821019	0.840777
H	3.748709	0.356799	1.499563
H	2.333973	0.919503	-0.485262
H	2.675017	-0.605715	-2.285532
H	0.444907	-1.421193	-0.978093
H	-0.095798	1.389476	0.088166
H	-2.409537	0.540067	0.411104
H	-1.952770	-0.999921	-0.352292
H	-1.173908	0.535531	-2.112452

α-D-glucose$^{-}_{(aq)}$ (C6-O^{-})

C	-0.292938	0.321531	0.031741
O	-0.126867	-0.329227	1.302973
C	1.146219	-0.204180	1.892103
C	2.230062	-0.750232	0.958160
C	2.130421	-0.109117	-0.424877
C	0.713420	-0.254562	-0.964798
O	1.470339	1.134042	2.195797
O	3.523082	-0.597640	1.520955
O	3.015870	-0.736370	-1.347224
O	0.561749	0.447730	-2.192833
C	-1.759040	0.150568	-0.385761
O	-2.169684	-1.160034	-0.604417
H	1.098959	-0.796572	2.809393
H	0.976579	1.401062	2.984402
H	2.070572	-1.827338	0.857033
H	3.647896	0.341435	1.729540
H	2.376140	0.959783	-0.354929
H	3.923820	-0.523449	-1.088407
H	0.499807	-1.321332	-1.118806
H	1.191685	0.074801	-2.826262
H	-0.094437	1.396160	0.152790
H	-1.887733	0.794001	-1.280087
H	-2.344911	0.646467	0.417519

α-D-glucose$^{2-}_{(aq)}$ (C1-O^{-}, C4-O^{-})

C	-0.285331	0.306441	0.005615
C	-0.059463	-0.295171	1.403091
O	1.302833	-0.150814	1.792048
C	1.759299	1.234969	1.911901
C	1.547333	1.940543	0.545186
C	0.114906	1.781010	0.063210
C	-0.382399	-1.785038	1.419587
O	-1.741827	-1.998134	1.050314
O	1.184100	1.920720	2.922215
O	1.899473	3.315581	0.708780
O	-0.031082	2.373365	-1.230483
O	-1.595803	0.147831	-0.454900

β-D-glucose$^{0}_{(aq)}$

C	-0.312660	0.278704	0.048984
O	-0.131520	-0.437829	1.269277
C	1.130760	-0.208603	1.875298
C	2.244854	-0.705564	0.956228
C	2.131559	-0.024295	-0.402959
C	0.726712	-0.190283	-0.972725
O	3.522362	-0.397911	1.489997
O	3.042619	-0.577346	-1.343372
O	0.554668	0.590033	-2.145986
C	-1.750241	0.056590	-0.390593
O	-2.047856	-1.304213	-0.674332
H	2.131884	-1.790894	0.831817

β-D-glucose$^{-}_{(aq)}$ (C1-O^{-})

C	-0.302000	0.308297	0.031575
C	-0.084901	-0.449916	1.345622
O	1.216982	-0.191342	1.847642
C	1.454140	1.218977	2.185512
C	1.319500	2.003322	0.866474
C	-0.044815	1.795908	0.233418
C	-0.227768	-1.957089	1.201036
O	0.684578	-2.517759	0.262678
O	2.655896	1.362929	2.758870
O	1.492104	3.396116	1.110332
O	-0.153214	2.419636	-1.046308
O	-1.638557	0.071850	-0.394851

H	2.846454	1.087502	2.055355
H	2.230760	1.526872	-0.204511
H	1.595385	3.524515	1.612636
H	-0.561456	2.296438	0.762766
H	-0.861466	2.002630	-1.575588
H	0.438500	-0.198878	-0.669984
H	-0.716215	0.215935	2.124016
H	-0.227717	-2.202427	2.420257
H	0.290159	-2.309388	0.723358
H	-1.917329	-1.279813	0.373674

β-D-glucose$^{-}_{(aq)}$ (C2-O^{-})

C	-0.089445	1.804229	0.251732
C	-0.336569	0.319193	0.034442
C	-0.100380	-0.439435	1.348153
O	1.200097	-0.150145	1.860398
C	1.389001	1.227747	2.129313
C	1.305187	2.050568	0.840422
O	-1.677923	0.051640	-0.366894
C	-0.194237	-1.949630	1.209758
O	0.716221	-2.485950	0.257534
O	2.664658	1.329367	2.713508
O	1.570773	3.393067	1.047679
O	-0.223855	2.490080	-0.990542
H	2.037500	1.587641	0.143098
H	-0.844372	2.188385	0.956668
H	0.199440	3.354142	-0.850303
H	0.358722	-0.052871	-0.729145
H	-1.821913	0.467724	-1.228533
H	-0.860178	-0.122353	2.079664
H	-1.195791	-2.222257	0.872091
H	-0.027198	-2.403592	2.194246
H	1.615423	-2.296036	0.561244
H	3.022523	2.183753	2.416753
H	0.623638	1.553960	2.852980

β-D-glucose$^{-}_{(aq)}$ (C6-O^{-})

C	-0.304109	0.275260	0.046003
C	-0.103403	-0.507393	1.345805
O	1.189508	-0.194884	1.878231
C	1.343320	1.169743	2.217595
C	1.235192	2.030887	0.960861
C	-0.102144	1.767967	0.280107
C	-0.186638	-2.029810	1.173458
O	0.701206	-2.569240	0.249918
O	2.622376	1.341183	2.758808
O	1.294865	3.413912	1.274913
O	-0.184249	2.416725	-0.983922
O	-1.621246	0.013403	-0.422486
H	2.053828	1.756678	0.282185
H	2.205419	3.634158	1.516507
H	-0.907724	2.134356	0.932644
H	-0.243625	3.371146	-0.836763
H	0.429502	-0.068819	-0.695169
H	-1.741168	0.495057	-1.253564
H	-0.871763	-0.193632	2.071492
H	-1.246681	-2.242856	0.924981
H	-0.038022	-2.444139	2.193465
H	2.614471	1.040814	3.679010
H	0.572773	1.455241	2.949120

β-D-glucose$^{-}_{(aq)}$ (C1-O^{-}, 1w)

C	-0.015544	1.818698	0.224998

H	3.689474	-0.985832	2.240085
H	2.335227	1.048920	-0.278979
H	3.937027	-0.293186	-1.108456
H	0.557886	-1.251576	-1.197688
H	1.179415	0.271721	-2.813302
H	-0.176455	1.356080	0.229664
H	-1.938007	0.615302	-1.309219
H	-2.419403	0.439030	0.389622
H	-1.957711	-1.809378	0.146249
O	1.175947	-0.943000	3.062448
H	0.699841	-0.456037	3.750548
H	1.245389	0.866291	2.076375

β-D-glucose$^{-}_{(aq)}$ (C3-O^{-})

C	-0.245661	0.307738	0.001540
C	-0.121490	-0.424004	1.336912
O	1.136659	-0.096778	1.937311
C	1.317039	1.287336	2.208076
C	1.245656	2.065868	0.900227
C	-0.098829	1.819407	0.204152
C	-0.180442	-1.938762	1.235237
O	0.804448	-2.486418	0.366165
O	2.538927	1.428527	2.877261
O	1.404577	3.452355	1.168477
O	-0.220376	2.538997	-0.975083
O	-1.517628	0.018436	-0.570011
H	2.063842	1.719103	0.248007
H	0.993394	3.898567	0.407481
H	-0.886059	2.112258	0.934654
H	0.546476	-0.039658	-0.677898
H	-1.675132	0.735130	-1.208055
H	-0.936949	-0.101333	2.002835
H	-1.150531	-2.234611	0.830380
H	-0.080415	-2.368895	2.239534
H	1.676281	-2.274998	0.729306
H	3.259954	1.287184	2.243789
H	0.545269	1.627830	2.909446

β-D-glucose$^{2-}_{(aq)}$ (C1-O^{-}, C4-O^{-})

C	-0.313640	0.256504	-0.019312
C	-0.117267	-0.432284	1.335001
C	1.320775	-0.151547	1.815752
O	2.271373	-0.601258	0.857769
C	2.169300	0.105247	-0.420717
C	0.761421	-0.174583	-0.991185
O	-1.089636	-0.034246	2.255697
C	1.609287	-0.866973	3.130342
O	0.701264	-0.429751	4.136870
O	3.148358	-0.287687	-1.252706
O	0.611275	0.531316	-2.222845
O	-1.609119	-0.054059	-0.537195
H	1.519068	-1.953329	2.977918
H	0.681257	-1.256311	-1.178001
H	1.444240	0.391687	-2.703463
H	-0.244842	1.346028	0.131300
H	-2.199795	-0.012974	0.234437
H	-0.170287	-1.526123	1.147527
H	-0.149309	-0.268018	3.632675
H	1.438172	0.934816	1.971559
H	2.628705	-0.653017	3.468256
H	2.225399	1.188027	-0.163348

β-D-glucose$^{-}_{(aq)}$ (C2-O^{-}, 1w)

C	1.120534	2.140223	1.528917

H	2.101826	1.647177	0.179302
H	2.291717	3.476708	1.654747
H	-0.818337	2.201761	0.901012
H	-0.165362	3.378588	-0.919301
H	0.401000	-0.070590	-0.722652
H	-1.784690	0.581212	-1.205369
H	-0.842080	-0.112931	2.072824
H	-1.230081	-2.202266	0.844269
H	-0.088730	-2.418767	2.186537
H	1.582023	-2.311500	0.561803
H	0.609069	1.505506	2.852471

β-D-glucose$^{-}_{(aq)}$ (C4-O^{-})

C	-0.328029	0.286681	-0.030576
C	-0.074106	-0.379632	1.333891
O	1.259945	-0.106855	1.773027
C	1.502242	1.274031	1.985375
C	1.319228	2.054814	0.685470
C	-0.072358	1.786998	0.132570
C	-0.224247	-1.893073	1.248502
H	0.517610	-2.290040	0.539278
O	2.826850	1.413835	2.410674
O	1.520412	3.431665	0.982792
O	-0.203758	2.456724	-1.118220
O	-1.605680	0.032372	-0.526505
H	2.075996	1.711498	-0.033477
H	1.401830	3.932555	0.162976
H	-0.815203	2.193266	0.837887
H	-0.996035	2.074590	-1.531350
H	0.454847	-0.101906	-0.715832
H	-1.785562	-1.488106	0.194228
H	-0.789507	0.014684	2.073314
O	-1.543028	-2.223645	0.828810
H	-0.046304	-2.354608	2.224988
H	2.878576	1.165020	3.344737
H	0.808606	1.645732	2.753946

β-D-glucose$^{0}_{(aq)}$ (1w)

C	0.750966	-0.298279	-1.138395
C	-0.288447	0.227417	-0.144577
O	-0.101298	-0.410683	1.116665
C	1.164131	-0.135686	1.705415
C	2.273566	-0.690199	0.813679
C	2.157179	-0.093708	-0.583781
C	-1.725557	-0.027961	-0.567193
O	-2.013105	-1.403015	-0.785925
O	1.212566	-0.772113	2.941698
O	3.554114	-0.354466	1.323325
O	3.067031	-0.700796	-1.492558
O	0.573940	0.405093	-2.358677
H	2.157821	-1.781109	0.756779
H	3.696625	-0.856035	2.138678
H	2.359104	0.985341	-0.526478
H	3.959977	-0.390613	-1.286656
H	0.586110	-1.372224	-1.297126
H	1.202003	0.048418	-3.003269
H	-0.157872	1.314730	-0.030297
H	-1.922381	0.484779	-1.510482
H	-2.394843	0.385211	0.197303
H	-1.901822	-1.872768	0.053015
H	0.737993	-0.203650	3.591282
H	1.272044	0.953825	1.822976
O	-0.152277	0.966071	4.576135
H	-1.006345	1.024785	4.123612
H	0.278514	1.811871	4.384037

β-D-glucose$^{-}_{(aq)}$ (C3-O^{-}, 1w)

C	0.994247	1.965909	0.312535

C	-0.158570	0.313808	0.038039
C	-0.052266	-0.401079	1.389693
O	1.157660	-0.044255	2.038591
C	1.281826	1.378544	2.345575
C	1.254572	2.123205	0.998506
O	-1.419739	-0.016535	-0.530257
C	-0.055649	-1.917383	1.271662
O	1.034769	-2.412010	0.501281
O	2.416435	1.595183	3.036166
O	1.320683	3.528614	1.215662
O	-0.015297	2.409296	-1.073933
H	2.127261	1.796702	0.413319
H	2.072950	3.677706	1.810145
H	-0.882499	2.189416	0.790720
H	-0.077298	3.369408	-0.972522
H	0.648038	-0.036667	-0.620065
H	-1.482536	0.425104	-1.389352
H	-0.914979	-0.107805	2.010189
H	-0.967737	-2.253715	0.775136
H	-0.031246	-2.347436	2.280575
H	1.852648	-2.085660	0.905471
H	0.369491	1.648239	2.919879
H	3.649160	0.850268	2.211577
O	4.400058	0.382949	1.733652
H	3.955419	-0.294499	1.205551

β-D-glucose$^{-}_{(aq)}$ (C4-O^{-}, 1w)

C	0.341210	1.674265	-0.245426
C	0.066020	0.166861	-0.287845
C	-0.115630	-0.330254	1.162010
O	1.028960	-0.017842	1.957988
C	1.222313	1.379991	2.083459
C	1.483350	1.998722	0.711496
O	-1.039860	-0.162076	-1.073035
C	-0.297871	-1.842816	1.216646
O	-1.461067	-2.227141	0.493972
O	2.348379	1.592627	2.885631
O	1.583738	3.413975	0.803520
O	0.649384	2.117822	-1.563336
H	0.595825	-2.329666	0.798443
H	2.418310	1.578067	0.316860
H	2.415408	3.634165	1.246637
H	-0.567253	2.187282	0.105757
H	0.748878	3.080120	-1.538255
H	0.984465	-0.317456	-0.675513
H	-1.489716	-1.583614	-0.269414
H	-1.003357	0.153806	1.599194
H	-0.412744	-2.171593	2.254311
H	2.093688	1.490489	3.813977
H	0.330327	1.828307	2.545272
H	-2.358158	0.641427	-0.585311
O	-3.209493	1.119495	-0.320361
H	-2.956523	2.051299	-0.269320

β-D-glucose$^{0}_{(aq)}$ (2w)

C	2.139205	-0.131686	-0.470685
C	0.706471	-0.355907	-0.944869
C	-0.287566	0.282177	0.027557
O	-0.047859	-0.227495	1.343957
C	1.246937	0.096108	1.848099
C	2.305638	-0.576120	0.977780
O	0.491938	0.227308	-2.220806
C	-1.747892	0.025719	-0.325145
O	-2.056528	-1.314249	-0.676707
O	1.335271	-0.394342	3.144982
O	3.612772	-0.215647	1.393365
O	2.992069	-0.861158	-1.343903
O	0.054753	1.582009	4.555753
H	2.166595	-1.664029	1.046901

C	-0.216387	1.960579	0.803513
C	-0.305384	0.580345	0.168499
C	-0.041401	-0.498908	1.227914
O	1.207673	-0.257453	1.874792
C	1.245761	0.994365	2.533847
O	-0.370960	2.941866	-0.218634
O	-1.596365	0.325327	-0.376690
C	0.016677	-1.908981	0.665015
O	1.004400	-2.075370	-0.344514
O	2.484559	1.040346	3.197680
O	1.243412	3.377774	2.141700
H	1.923861	1.978077	0.779059
H	-1.033993	2.069174	1.533710
H	-0.098243	3.787585	0.172396
H	0.452292	0.502581	-0.622377
H	-1.762073	0.978275	-1.071286
H	-0.850789	-0.468863	1.973945
H	-0.942326	-2.154674	0.205311
H	0.193770	-2.609242	1.490306
H	1.872163	-1.916393	0.053390
H	2.746908	1.977303	3.199040
H	0.426974	1.028821	3.271332
H	2.016877	4.325054	1.114909
O	2.514916	4.917410	0.458931
H	2.202767	4.630659	-0.409824

β-D-glucose$^{-}_{(aq)}$ (C6-O^{-}, 1w)

C	0.036752	1.628648	0.264739
C	-0.254521	0.142481	0.094968
C	-0.181103	-0.583628	1.440200
O	1.091666	-0.320958	2.042342
C	1.314567	1.047538	2.319983
C	1.344372	1.841808	1.016249
O	-1.558944	-0.054673	-0.436228
C	-0.352414	-2.103543	1.333796
O	0.498103	-2.741895	0.435116
O	2.565068	1.165509	2.936342
O	1.481641	3.232695	1.261439
O	0.078302	2.206645	-1.035277
H	2.182307	1.476327	0.406915
H	2.391628	3.405280	1.541456
H	-0.780670	2.084932	0.841294
H	0.043007	3.169195	-0.944887
H	0.492490	-0.287851	-0.584840
H	-1.610485	0.427599	-1.274194
H	-0.970970	-0.190284	2.100878
H	-1.417227	-2.270876	1.083141
H	-0.228971	-2.483830	2.368184
H	2.478191	0.921461	3.869227
H	0.519912	1.421472	2.982885
H	1.946756	-2.086157	0.223088
O	2.869512	-1.691090	0.060193
H	2.834501	-0.871766	0.574972

β-D-glucose$^{-}_{(aq)}$ (C1-O^{-}, 2w)

C	1.335986	2.192223	0.815342
C	0.019546	1.865158	0.133429
C	-0.176117	0.355063	0.085413
C	-0.024039	-0.246655	1.486811
O	1.222529	0.128162	2.052361
C	1.406513	1.561599	2.215180
O	-0.028784	2.341989	-1.209863
O	-1.474565	0.019223	-0.386631
C	-0.064190	-1.767077	1.499779
O	0.991101	-2.350609	0.742986
O	2.589412	1.792014	2.822307
O	1.450991	3.607565	0.909462
O	4.396031	0.302331	1.491358
H	2.167827	1.789338	0.218958

C	-0.339688	1.420690	-0.227521
C	-0.362727	-0.097836	-0.018395
C	-0.117366	-0.459804	1.441184
O	1.151319	0.074788	1.827394
C	1.223541	1.489805	1.748793
O	-0.578365	1.712156	-1.568297
O	-1.624897	-0.610683	-0.429687
C	-0.081651	-1.950724	1.730091
O	0.876906	-2.658689	0.952912
O	2.470016	1.873308	2.258463
O	1.031803	3.386772	0.351649
O	-0.169033	4.184878	-2.077215
H	1.816220	1.585055	-0.315312
H	0.706620	3.738881	-0.503541
H	-1.135175	1.846167	0.420637
H	0.431787	-0.546357	-0.632749
H	-1.848745	-0.098262	-1.226127
H	-0.907629	-0.017801	2.068056
H	-1.055031	-2.386309	1.495123
H	0.112222	-2.100473	2.799464
H	1.756868	-2.334454	1.191895
H	3.154526	1.650812	1.607915
H	0.472927	1.928029	2.418780
H	-0.413659	3.199306	-1.918527
H	-0.939934	4.692159	-1.788269

β-D-glucose$^{2-}_{(aq)}$ (C1-O^{-}, C4-O^{-}, 1w)

C	0.871240	-0.286333	-0.859782
C	-0.254980	0.220872	0.011864
C	-0.137401	-0.371128	1.419556
C	1.266884	-0.044738	1.966033
O	2.274635	-0.556959	1.100547
C	2.240064	0.046042	-0.226733
O	-1.521739	-0.124470	-0.550223
O	-1.168100	0.077138	2.247809
C	1.476140	-0.661731	3.343938
O	0.504010	-0.161665	4.257042
O	3.274902	-0.399680	-0.966912
O	0.793877	0.311990	-2.152429
H	1.401386	-1.756961	3.264735
H	0.793812	-1.379143	-0.958167
H	1.639081	0.111646	-2.587054
H	-0.187183	1.318352	0.086422
H	-2.145712	-0.045769	0.192309
H	-0.171886	-1.475609	1.303768
H	-0.314427	-0.042835	3.690880
H	1.373317	1.050596	2.049028
H	2.470667	-0.413843	3.729310
H	2.284721	1.144225	-0.060078
H	3.047233	-1.867015	-1.662135
O	2.972948	-2.758167	-2.126859
H	2.048394	-2.809569	-2.405297

β-D-glucose$^{-}_{(aq)}$ (C2-O^{-}, 2w)

O	1.597041	0.967748	2.441879
C	1.565633	2.058088	1.524072
C	0.193775	2.142232	0.834195
C	-0.878173	2.268592	1.927895
C	-0.741656	1.167324	2.971716
C	0.683981	1.129738	3.522960
O	2.622325	1.849055	0.627716
O	0.156561	3.182956	-0.090959
O	-2.198246	2.172740	1.399500
O	-1.630740	1.380064	4.062630
C	0.939029	0.001287	4.508523
O	0.667958	-1.289549	3.975852
O	-2.080363	4.526006	-0.147067
O	1.479655	1.986895	-1.976081

H	3.784088	-0.628974	2.251794
H	2.369461	0.940707	-0.541840
H	3.899978	-0.551716	-1.217518
H	0.517743	-1.436990	-0.989313
H	1.088833	-0.203920	-2.849235
H	-0.136539	1.371910	0.040893
H	-2.010339	0.637305	-1.191087
H	-2.362325	0.356772	0.522216
H	-1.781909	-1.913403	0.045502
H	0.897691	0.257970	3.740449
H	1.365795	1.189864	1.830294
H	-0.787817	1.612935	4.079312
H	0.525478	2.374439	4.258833
H	-0.357156	-2.055304	1.484094
O	-0.801400	-2.915620	1.340074
H	-0.200139	-3.400458	0.756943

β-D-glucose$^{-}_{(aq)}$ (C3-O^{-}, 2w)

O	1.273510	1.864668	1.786909
C	1.421290	2.198204	0.417564
C	0.271451	1.595980	-0.384237
C	-1.094469	2.049612	0.158532
C	-1.146198	1.827744	1.679853
C	0.089742	2.407672	2.370788
O	2.676087	1.736953	0.001777
O	0.461167	1.973927	-1.743060
O	-2.130105	1.387824	-0.506353
O	-2.272722	2.468870	2.268653
C	0.159219	2.124488	3.862325
O	0.118941	0.737757	4.179307
O	-2.183505	2.465844	-2.846623
O	-4.448225	1.710439	0.609650
H	0.334764	0.499647	-0.292693
H	-0.407406	2.060776	-2.183359
H	-1.158222	3.145402	-0.003481
H	-1.191353	0.745698	1.869708
H	-3.085043	2.168272	1.812250
H	0.090525	3.501391	2.239515
H	-0.699492	2.581097	4.357615
H	1.072051	2.579674	4.265870
H	0.907392	0.319036	3.805604
H	2.645132	0.769461	-0.063792
H	1.444211	3.289634	0.305241
H	-2.282677	2.059225	-1.918650
H	-2.220215	3.422254	-2.708181
H	-3.586645	1.606093	0.084169
H	-4.811846	2.564596	0.339256

H	2.242592	3.786453	1.440751
H	-0.805621	2.311383	0.706750
H	-0.087901	3.307225	-1.187623
H	0.584345	-0.076302	-0.579681
H	-1.580536	0.406398	-1.267431
H	-0.850015	0.121506	2.116531
H	-0.998085	-2.123285	1.061198
H	-0.017074	-2.109082	2.541137
H	1.828474	-2.018079	1.099628
H	0.544084	1.921136	2.814731
H	3.742900	0.873520	1.986479
H	3.837220	-0.351992	1.048914
H	2.346073	1.935832	4.432952
O	2.180136	2.067238	5.415356
H	2.156705	1.174565	5.784770

β-D-glucose$^{-}_{(aq)}$ (C4-O^{-}, 2w)

C	1.400851	1.919181	1.364182
C	0.132931	1.598927	0.577327
C	0.140082	0.146336	0.109882
C	1.437905	-0.195322	-0.630884
C	2.632204	0.251680	0.238820
O	2.540773	1.636813	0.570910
O	-1.027030	1.784873	1.377634
O	-0.962676	-0.091806	-0.761090
O	1.554371	-1.553049	-0.937711
C	3.960619	0.059562	-0.484337
O	4.181880	-1.315268	-0.774213
O	1.407058	3.286249	1.659515
O	1.597781	-3.045663	1.254484
O	-0.568519	-2.791215	-1.926715
H	3.957528	0.652420	-1.411060
H	0.098359	2.261019	-0.298609
H	-1.172167	2.734187	1.494113
H	0.057374	-0.509399	0.989605
H	-1.776445	0.060476	-0.259481
H	1.458333	0.420329	-1.550939
H	3.276419	-1.667178	-0.981533
H	2.640168	-0.342651	1.166519
H	4.782991	0.411818	0.145709
H	2.035080	3.446315	2.378803
H	1.445951	1.324449	2.288406
H	0.239573	-2.318056	-1.566376
H	-1.277861	-2.171871	-1.701817
H	1.565655	-2.460802	0.436897
H	2.313736	-2.670434	1.785088

H	0.037164	1.160471	0.341443
H	-0.756995	3.242988	2.426012
H	-2.375861	2.991043	0.892535
H	-0.965089	0.202672	2.495747
H	-2.517650	1.498085	3.691083
H	0.891740	2.078819	4.042240
H	0.286659	0.123607	5.375310
H	1.979846	0.062047	4.849379
H	1.287744	-1.453299	3.250972
H	2.274518	1.702025	-0.277105
H	1.769784	2.982809	2.085031
H	-1.188774	4.048848	-0.189940
H	-2.517574	4.322297	-0.985282
H	0.878273	2.517883	-1.355205
H	0.981970	1.184294	-2.184065

β-D-glucose$^{-}_{(aq)}$ (C6-O^{-}, 2w)

O	1.213464	1.781645	2.233687
C	0.744624	2.260036	0.987863
C	-0.541031	1.531281	0.603962
C	-1.576403	1.730139	1.703335
C	-1.004271	1.332221	3.058356
C	0.321339	2.052458	3.320063
O	1.716492	1.979523	0.021808
O	-1.084691	2.044868	-0.601677
O	-2.738710	0.938989	1.486797
O	-1.914888	1.674122	4.099220
C	1.018278	1.638631	4.620039
O	1.240674	0.270686	4.770611
O	-1.011494	-0.504502	5.867144
O	1.460936	-1.210353	2.599228
H	-0.317277	0.460998	0.496831
H	-0.507184	1.780845	-1.332043
H	-1.857478	2.792482	1.735829
H	-3.255184	1.339620	0.773921
H	-0.830730	0.248109	3.063276
H	-2.784595	1.328289	3.846607
H	0.132835	3.137504	3.362479
H	0.394768	2.039534	5.441121
H	1.962827	2.212711	4.649227
H	2.418446	2.643883	0.082595
H	0.567750	3.343839	1.056003
H	-0.113718	-0.273344	5.459469
H	-1.589123	0.165588	5.464365
H	1.343962	-0.569556	1.882882
H	1.364929	-0.632515	3.421715

β-D-glucose$^{2-}_{(aq)}$ (C1-O^{-}, C4-O^{-}, 2w)

O	1.756011	1.961166	1.726688
C	0.554820	2.118283	0.910350
C	0.244990	0.727146	0.316986
C	0.065492	-0.320238	1.406625
C	1.276479	-0.364833	2.337806
C	1.579639	1.069130	2.817862
O	0.771993	3.079510	-0.013048
O	-0.954745	0.744099	-0.456635
O	-0.118082	-1.619316	0.839043
O	1.097551	-1.229216	3.421977
C	2.861136	1.111045	3.645413
O	2.754528	0.271641	4.790477
O	2.192364	-3.501473	2.766637
H	3.705290	0.792633	3.015027
H	1.090043	0.439763	-0.326753
H	-0.751726	1.213907	-1.289855
H	-0.824574	-0.062398	2.002681
H	-0.834869	-1.551074	0.191428
H	2.142826	-0.681064	1.719626
H	2.175340	-0.481184	4.478985
H	0.738865	1.412718	3.444511

H	3.056295	2.133492	3.985139
H	-0.258588	2.384881	1.619720
H	1.758030	-2.619355	3.019427
H	1.863375	-3.686748	1.876803
H	0.542510	2.657584	-1.549385
O	0.248547	2.340193	-2.464300
H	0.958341	1.758139	-2.769055

Sample inputs for calculations of pK_a values *via* thermodynamic cycle using Gaussian 09 (revision D.01)

```
%mem=24GB
%nProc=4
#p CAM-B3LYP/aug-cc-pVTZ nosymm opt freq

text

-1,1
 C   -0.341240    0.306568    0.050671
 C   -0.097323   -0.458165    1.350234
 O    1.199211   -0.210898    1.846571
 C    1.452838    1.228192    2.158488
 C    1.316800    1.954626    0.813743
 C   -0.058964    1.781336    0.235872
 C   -0.201080   -1.959226    1.166967
 O    0.866173   -2.451645    0.372849
 O    2.610235    1.435415    2.718883
 O    1.598810    3.327993    1.024716
 O   -0.222948    2.399836   -1.038725
 O   -1.687394    0.095033   -0.347952
 H    2.057677    1.519684    0.131842
 H    2.268487    3.289641    1.728360
 H   -0.795219    2.195011    0.934089
 H   -0.047902    3.339744   -0.934004
 H    0.339279   -0.081819   -0.713611
 H   -1.866733    0.709703   -1.067119
 H   -0.860947   -0.142020    2.074477
 H   -1.131563   -2.215958    0.665073
 H   -0.193924   -2.433452    2.152735
 H    1.655707   -2.000552    0.696279
 H    0.568320    1.506781    2.787205
```

```
%mem=24GB
%nProc=4
#p CAM-B3LYP/aug-cc-pVTZ nosymm scrf=(pcm,solvent=water)

text

-1,1
 C   -0.363143    0.306333    0.048537
 C   -0.095375   -0.468042    1.342377
 O    1.197024   -0.222704    1.827638
 C    1.456468    1.244829    2.159224
 C    1.298808    1.946656    0.797883
 C   -0.088746    1.778168    0.254762
 C   -0.191050   -1.972582    1.145382
 O    0.938752   -2.460036    0.444454
 O    2.601600    1.475181    2.698035
 O    1.601476    3.321542    0.969737
 O   -0.291106    2.451461   -0.994132
 O   -1.717072    0.093428   -0.346245
 H    2.025636    1.490299    0.117030
 H    2.271814    3.278708    1.677641
 H   -0.807926    2.172769    0.981366
 H    0.146707    3.304217   -0.911987
 H    0.307488   -0.069681   -0.729592
 H   -1.907604    0.744737   -1.027616
 H   -0.861021   -0.162567    2.071819
 H   -1.080334   -2.228433    0.571160
 H   -0.255823   -2.447640    2.130335
 H    1.676133   -1.932697    0.784779
 H    0.565593    1.491883    2.795056
```

Cartesian coordinates of structures used to calculate pK_a values *via* a thermodynamic cycle. Optimization was performed on the CAM-B3LYP/aug-cc-pVTZ level of theory. Minima were confirmed by the absence of imaginary vibrational frequencies.

α-D-glucose$^{0}_{(g)}$

O	-0.291760	0.362074	0.042125
C	-0.120800	-0.270327	1.276921
C	1.298529	-0.102617	1.804258
C	1.694801	1.362833	1.800415
C	1.489490	1.962297	0.426866
C	0.049922	1.753175	-0.017301
H	-0.339337	-1.322944	1.094276
O	-0.979979	0.243088	2.269673
H	-1.886541	-0.003070	2.068282
H	1.974916	-0.648582	1.146799
O	1.446755	-0.669225	3.089950
H	0.677125	-0.411555	3.610280
H	1.066480	1.907528	2.513535
O	3.059339	1.525576	2.138685
H	3.224803	1.056334	2.962345
H	2.157850	1.464538	-0.282343
O	1.730245	3.354161	0.436037
H	2.601506	3.500275	0.816901
H	-0.606517	2.325344	0.643481
C	-0.203047	2.179605	-1.447380
H	0.099318	3.217092	-1.565493
H	-1.274595	2.101707	-1.653246
O	0.542966	1.420943	-2.375324
H	0.324275	0.494411	-2.240405

α-D-glucose$^{-}_{(g)}$ (C1-O^{-})

C	-0.285241	0.330467	0.021166
O	-0.136562	-0.357315	1.238380
C	1.212064	-0.198313	1.898428
C	2.236278	-0.782261	0.893945
C	2.076055	-0.113014	-0.455404
C	0.680837	-0.255214	-1.003084
O	1.545884	1.014113	2.231146
O	3.533216	-0.479640	1.392905
O	3.002065	-0.637984	-1.415638
O	0.552067	0.438128	-2.242821
C	-1.742272	0.178913	-0.375684
O	-2.166586	-1.170083	-0.255666
H	1.071833	-0.912171	2.737571
H	2.147571	-1.866281	0.791532
H	3.330136	0.341182	1.894534
H	2.275465	0.952858	-0.315433
H	3.853440	-0.651482	-0.965813
H	0.444348	-1.314056	-1.151443
H	1.366390	0.274377	-2.728024
H	-0.054947	1.394034	0.146522
H	-1.887467	0.485498	-1.410491
H	-2.348185	0.819826	0.273392
H	-1.702133	-1.493850	0.528364

α-D-glucose$^{-}_{(g)}$ (C2-O^{-})

C	-0.287685	0.362588	-0.030937
O	-0.137363	-0.214183	1.258991
C	1.160988	-0.236991	1.842348
C	2.264975	-0.729376	0.886209
C	2.056013	-0.064635	-0.476457
C	0.675851	-0.278017	-1.022798
O	1.554771	1.023204	2.288960
O	3.493648	-0.397068	1.356504
O	3.049663	-0.545806	-1.370653
O	0.505528	0.347897	-2.295267
C	-1.749057	0.168892	-0.383770
O	-2.154466	-1.178529	-0.213821
H	1.038865	-0.921310	2.687604
H	2.534895	0.945047	2.134715
H	2.112048	-1.819992	0.739064
H	2.200712	1.013852	-0.340619
H	3.818284	-0.622078	-0.776937
H	0.457298	-1.346197	-1.112225
H	1.345151	0.242390	-2.753462
H	-0.073309	1.435375	0.003476
H	-1.911895	0.436938	-1.425874
H	-2.354288	0.825251	0.250586
H	-1.707815	-1.477419	0.587499

α-D-glucose$^{-}_{(g)}$ (C3-O^{-})

C	-0.260874	0.359277	0.018921
O	-0.081032	-0.251153	1.313491
C	1.226365	-0.218627	1.871446
C	2.224450	-0.781358	0.874609
C	2.136704	-0.046007	-0.463278
C	0.713259	-0.242733	-0.976259
O	1.574786	1.086947	2.254543
O	3.556542	-0.740806	1.322076
O	3.085717	-0.450372	-1.333085
O	0.607729	0.386515	-2.239436
C	-1.725669	0.158558	-0.308116
O	-2.126909	-1.195128	-0.179019
H	1.146592	-0.853456	2.759008
H	0.932137	1.374564	2.907546
H	1.925025	-1.825981	0.707477
H	4.024796	-0.682193	0.462638
H	2.218682	1.040048	-0.227331
H	0.512791	-1.315772	-1.081701
H	1.521515	0.288943	-2.570196
H	-0.063351	1.432869	0.086707
H	-1.899758	0.461126	-1.339329
H	-2.333067	0.788974	0.352455
H	-1.638561	-1.540687	0.576267

α-D-glucose$^{-}_{(g)}$ (C4-O^{-})

C	-0.269236	0.329263	0.044591
O	-0.071182	-0.232663	1.360189
C	1.235601	-0.203272	1.834412
C	2.293541	-0.749818	0.864638
C	2.101482	-0.137066	-0.503326
C	0.655225	-0.348173	-0.963261
O	1.633551	1.125223	2.165969
O	3.584236	-0.567377	1.429972
O	2.946638	-0.708121	-1.478405
O	0.486735	0.077208	-2.245002
C	-1.721530	0.128225	-0.342609
O	-1.944187	0.646392	-1.627114
H	1.228448	-0.808342	2.744741
H	0.906721	1.520620	2.653812
H	2.157412	-1.827716	0.784480
H	3.684969	0.375391	1.591736
H	2.285995	0.944455	-0.444309
H	2.412836	-0.566104	-2.285292
H	0.459109	-1.441639	-0.850241
H	-0.052578	1.401180	0.068479
H	-2.382217	0.629632	0.373047
H	-1.943785	-0.948313	-0.292290
H	-1.071369	0.476131	-2.115510

α-D-glucose$^{2-}_{(g)}$ (C1-O^{-}, C4-O^{-})

C	-0.258427	0.295426	0.000180
C	-0.058919	-0.270234	1.407505
O	1.297332	-0.146601	1.788405
C	1.803100	1.235989	1.876848
C	1.519386	1.996156	0.528810
C	0.098044	1.776776	0.054798
C	-0.441089	-1.741859	1.441316
O	-1.753411	-1.937895	0.960345
O	1.367400	1.946215	2.900691
O	1.786911	3.366369	0.822861
O	-0.157056	2.317739	-1.246925
O	-1.516602	0.103584	-0.537329
H	2.899674	1.025307	1.908795
H	2.210975	1.667424	-0.253116
H	1.615094	3.338331	1.796850
H	-0.575025	2.259333	0.772879
H	-0.900504	1.744828	-1.517586
H	0.527473	-0.193080	-0.621046
H	-0.703687	0.276936	2.105931
H	-0.363092	-2.127049	2.465733
H	0.293063	-2.294298	0.832374
H	-1.861528	-1.184709	0.270224

β-D-glucose$^{0}_{(g)}$

C	-0.319591	0.304379	0.039395
O	-0.129199	-0.386134	1.271862
C	1.141679	-0.196756	1.848825
C	2.223242	-0.714369	0.918413
C	2.112277	-0.012194	-0.416135
C	0.714236	-0.152736	-0.982231
O	3.517832	-0.458718	1.423657
O	3.012709	-0.541173	-1.369249
O	0.554174	0.649519	-2.133301
C	-1.744222	0.021223	-0.388999
O	-1.997349	-1.361098	-0.516949
H	2.062059	-1.788425	0.784979

β-D-glucose$^{-}_{(g)}$ (C1-O^{-})

C	-0.363143	0.306333	0.048537
C	-0.095375	-0.468042	1.342377
O	1.197024	-0.222704	1.827638
C	1.456468	1.244829	2.159224
C	1.298808	1.946656	0.797883
C	-0.088746	1.778168	0.254762
C	-0.191050	-1.972582	1.145382
O	0.938752	-2.460036	0.444454
O	2.601600	1.475181	2.698035
O	1.601476	3.321542	0.969737
O	-0.291106	2.451461	-0.994132
O	-1.717072	0.093428	-0.346245

β-D-glucose$^{-}_{(g)}$ (C2-O^{-})

C	-0.107114	1.770758	0.265256
C	-0.365528	0.305131	0.032463
C	-0.121097	-0.461570	1.337203
O	1.156424	-0.188077	1.893225
C	1.381142	1.204116	2.111599
C	1.310292	1.964906	0.792215
O	-1.713737	0.050068	-0.370099
C	-0.166624	-1.966942	1.162362
O	0.959782	-2.441237	0.448243
O	2.641891	1.374471	2.665895
O	1.674320	3.256888	0.892610
O	-0.260824	2.530121	-0.919884

H	3.615295	-0.915226	2.264252
H	2.320176	1.053996	-0.264266
H	3.900803	-0.501961	-1.001688
H	0.532555	-1.204646	-1.219885
H	1.255491	0.422941	-2.751552
H	-0.205420	1.384916	0.191140
H	-1.919447	0.471097	-1.363076
H	-2.425304	0.476953	0.335696
H	-1.766259	-1.781030	0.317015
O	1.215623	-0.931017	3.025128
H	0.605637	-0.567077	3.672636
H	1.294772	0.874258	2.043311

H	2.025636	1.490299	0.117030
H	2.271814	3.278708	1.677641
H	-0.807926	2.172769	0.981366
H	0.146707	3.304217	-0.911987
H	0.307488	-0.069681	-0.729592
H	-1.907604	0.744737	-1.027616
H	-0.861021	-0.162567	2.071819
H	-1.080334	-2.228433	0.571160
H	-0.255823	-2.447640	2.130335
H	1.676133	-1.932697	0.784779
H	0.565593	1.491883	2.795056

H	1.974864	1.379670	0.109743
H	-0.818864	2.138672	1.018510
H	0.362817	3.264515	-0.750862
H	0.316612	-0.064322	-0.736980
H	-1.938584	0.744611	-0.996200
H	-0.911270	-0.167346	2.041223
H	-1.057748	-2.245468	0.603113
H	-0.208526	-2.434372	2.152268
H	1.707402	-1.921820	0.769439
H	2.907904	2.228463	2.270723
H	0.607787	1.557991	2.812195

β-D-glucose$^{-}_{(g)}$ (C3-O^{-})

C	-0.269243	0.302622	-0.009309
C	-0.164648	-0.443552	1.309164
O	1.078016	-0.131903	1.950532
C	1.345736	1.250274	2.198473
C	1.271034	2.004322	0.889266
C	-0.106045	1.795204	0.254304
C	-0.185856	-1.954750	1.196121
O	1.001427	-2.462511	0.612890
O	2.588654	1.318868	2.827607
O	1.467828	3.390165	1.031048
O	-0.302245	2.573747	-0.824252
O	-1.531265	0.098554	-0.615710
H	2.040255	1.580906	0.221459
H	0.912221	3.719063	0.290536
H	-0.839424	2.024259	1.072536
H	0.525085	-0.038132	-0.683573
H	-1.646976	0.937149	-1.101046
H	-1.003645	-0.133123	1.943976
H	-1.021008	-2.251261	0.563712
H	-0.325046	-2.388594	2.193141
H	1.715984	-1.923241	0.970671
H	3.256971	1.431534	2.146373
H	0.611199	1.633678	2.911825

β-D-glucose$^{-}_{(g)}$ (C4-O^{-})

C	-0.297900	0.286782	-0.003054
C	-0.108195	-0.425646	1.343741
O	1.210796	-0.182659	1.858950
C	1.461855	1.171518	2.069396
C	1.352913	1.964092	0.771654
C	-0.033103	1.772503	0.227353
C	-0.305950	-1.919521	1.181818
H	0.488357	-2.301655	0.524472
O	2.752381	1.264459	2.616681
O	1.665756	3.327550	1.071309
O	-0.223123	2.467037	-0.990861
O	-1.512353	0.101671	-0.585586
H	2.089317	1.569233	0.066223
H	1.310793	3.854755	0.349583
H	-0.760312	2.139146	0.965119
H	-0.926412	1.926500	-1.402946
H	0.540811	-0.077999	-0.640761
H	-1.769632	-1.389786	0.048004
H	-0.852776	-0.045816	2.055526
O	-1.583751	-2.173975	0.655093
H	-0.203185	-2.426495	2.146048
H	3.056352	2.160784	2.440807
H	0.729686	1.574122	2.787934

β-D-glucose$^{-}_{(g)}$ (C6-O^{-})

C	-0.158458	0.307374	-0.063279
C	-0.222232	-0.373653	1.310738
O	0.924041	-0.052216	2.124888
C	1.043461	1.309214	2.354950
C	1.214167	2.053139	1.041426
C	0.049136	1.803143	0.107058
C	-0.331248	-1.893229	1.142020
O	-1.353054	-2.214253	0.297971
O	2.163995	1.521176	3.187706
O	1.329094	3.455125	1.265729
O	0.298254	2.424025	-1.146714
O	-1.330992	0.021661	-0.751165
H	2.127078	1.677632	0.565018
H	2.005620	3.577547	1.938380
H	-0.862986	2.222393	0.548075
H	0.516482	3.342111	-0.964382
H	0.727759	-0.086603	-0.590709
H	-1.502903	-0.984867	-0.457529
H	-1.121637	-0.013142	1.824666
H	-0.438646	-2.327149	2.158601
H	0.668873	-2.233163	0.786289
H	2.788466	0.822584	2.964059
H	0.177417	1.706909	2.899403

β-D-glucose$^{2-}_{(g)}$ (C1-O^{-}, C4-O^{-})

C	-0.320068	0.277855	-0.031785
C	-0.102165	-0.412460	1.317980
C	1.337477	-0.153430	1.788144
O	2.285706	-0.599349	0.841250
C	2.149144	0.109275	-0.451509
C	0.726325	-0.199220	-1.000278
O	-1.075239	-0.049047	2.226719
C	1.581455	-0.859460	3.112715
O	0.622299	-0.478337	4.076431
O	3.078162	-0.188436	-1.329983
O	0.669387	0.415026	-2.287211
O	-1.674133	0.019136	-0.414222
H	1.551825	-1.944490	2.927929
H	0.651824	-1.286695	-1.116339
H	1.615484	0.344734	-2.531784
H	-0.203294	1.360519	0.110336
H	-2.091308	-0.020514	0.467498
H	-0.138527	-1.503179	1.100288
H	-0.209876	-0.279115	3.510842
H	1.446092	0.930745	1.957332
H	2.587016	-0.623844	3.481960
H	2.148415	1.191749	-0.149986

β-D-glucose$^{0}_{(g)}$ (1w)

C	0.718277	-0.219561	-1.045423
C	-0.211472	0.376551	0.002833
O	0.037828	-0.209676	1.281265
C	1.380599	-0.062570	1.745956
C	2.335733	-0.720668	0.771196
C	2.160266	-0.113767	-0.599366
C	-1.672696	0.119798	-0.301878
O	-1.982332	-1.254467	-0.295979
O	1.510710	-0.691365	2.962559
O	3.678829	-0.520659	1.155705
O	2.952345	-0.761621	-1.573586
O	0.508177	0.495610	-2.243725
H	2.091800	-1.787361	0.737398
H	3.789116	-0.877504	2.042321
H	2.431155	0.948095	-0.545578
H	3.861917	-0.765437	-1.260232
H	0.465543	-1.275505	-1.177083
H	1.161676	0.189307	-2.879974
H	-0.038319	1.458964	0.043935
H	-1.896975	0.497455	-1.296584
H	-2.287998	0.665674	0.422043
H	-1.680453	-1.619843	0.541309
H	0.946620	-0.232021	3.607699
H	1.599576	1.012622	1.819816
O	-0.714215	0.616872	3.899950
H	-0.889318	0.430784	2.965880
H	-0.916655	1.544530	4.038730

β-D-glucose$^{-}_{(g)}$ (C1-O^{-}, 1w)

C	-0.041751	1.769197	0.239716
C	-0.243429	0.286529	0.039540
C	-0.108710	-0.443676	1.375648
O	1.114708	-0.143922	1.997973
C	1.297471	1.316734	2.304820
C	1.267073	2.003032	0.932383
O	-1.532702	0.021123	-0.502766
C	-0.136008	-1.953286	1.211993
O	1.063758	-2.415883	0.623609
O	2.387893	1.575782	2.964249
O	1.465222	3.395533	1.115485
O	-0.120469	2.407829	-1.036518
H	2.090579	1.582780	0.345168
H	2.091045	3.413938	1.858539
H	-0.851643	2.149935	0.874017
H	0.295308	3.268226	-0.924537
H	0.529225	-0.085832	-0.639622
H	-1.651493	0.649158	-1.221432
H	-0.959098	-0.141847	2.005413
H	-0.963563	-2.246901	0.567561
H	-0.277087	-2.407692	2.199204
H	1.750616	-1.863555	1.021086
H	0.359577	1.576453	2.854256
H	3.755406	1.150882	2.168286
O	4.543467	0.827442	1.639140
H	4.342214	-0.101885	1.517044

β-D-glucose$^{-}_{(g)}$ (C4-O^{-}, 1w)

C	0.036639	1.665670	-0.023567

β-D-glucose$^{2-}_{(g)}$ (C1-O^{-}, C4-O^{-}, 1w)

C	0.291422	-0.242132	-0.988486

C	-0.187422	0.147589	-0.094998
C	-0.036215	-0.426838	1.329349
O	1.198825	-0.053852	1.965211
C	1.311752	1.327516	2.085641
C	1.310813	1.988385	0.725981
O	-1.395708	-0.221747	-0.600158
C	-0.095237	-1.944224	1.306266
O	-1.293396	-2.390216	0.742104
O	2.535845	1.628190	2.707453
O	1.422580	3.404612	0.844761
O	0.116094	2.220409	-1.333596
H	0.785172	-2.303750	0.749594
H	2.164033	1.588688	0.168803
H	2.213795	3.581024	1.360779
H	-0.818939	2.105976	0.500290
H	0.363638	3.142345	-1.216185
H	0.669176	-0.250253	-0.684849
H	-1.532643	-1.653873	0.087841
H	-0.876312	-0.049591	1.926306
H	-0.001198	-2.324773	2.330252
H	2.591986	1.081117	3.495475
H	0.476977	1.720679	2.687900
H	-2.449709	1.009002	-0.956963
O	-2.912350	1.852508	-1.227879
H	-2.199613	2.281667	-1.708453

C	-0.339184	0.614838	0.075042
C	-0.307327	-0.133836	1.410566
C	1.141796	-0.528110	1.725898
O	1.695929	-1.323066	0.692746
C	1.737141	-0.612013	-0.582756
O	-1.698870	0.972337	-0.168217
O	-0.931449	0.591823	2.400748
C	1.195914	-1.301381	3.033397
O	0.603354	-0.563039	4.079269
O	2.329481	-1.310685	-1.548094
O	0.377353	0.392240	-2.263222
H	0.680706	-2.261974	2.879553
H	-0.269661	-1.178224	-1.086713
H	1.137845	-0.079646	-2.646841
H	0.249445	1.536226	0.180018
H	-2.007108	1.097334	0.749760
H	-0.829609	-1.098368	1.221068
H	-0.118052	-0.015484	3.601911
H	1.728395	0.395925	1.850197
H	2.236459	-1.531915	3.290679
H	2.269805	0.341565	-0.353385
H	3.826382	-1.391441	-1.502407
O	4.862020	-1.481944	-1.426747
H	4.969697	-1.763785	-0.516412

References

1. Thapa, B. and H.B. Schlegel, *Density Functional Theory Calculation of pKa's of Thiols in Aqueous Solution Using Explicit Water Molecules and the Polarizable Continuum Model.* The Journal of Physical Chemistry A, 2016. **120**(28): p. 5726-5735.
